# Omnipose: a high-precision morphology-independent solution for bacterial cell segmentation

**DOI:** 10.1038/s41592-022-01639-4

**Published:** 2022-10-17

**Authors:** Kevin J. Cutler, Carsen Stringer, Teresa W. Lo, Luca Rappez, Nicholas Stroustrup, S. Brook Peterson, Paul A. Wiggins, Joseph D. Mougous

**Affiliations:** 1 Department of Physics, University of Washington, Seattle, WA USA; 2 HHMI Janelia Research Campus, Ashburn, VA USA; 3 Centre for Genomic Regulation (CRG), The Barcelona Institute of Science and Technology, Barcelona, Spain; 4 Universitat Pompeu Fabra (UPF), Barcelona, Spain; 5 Department of Microbiology, University of Washington, Seattle, WA USA; 6 Department of Bioengineering, University of Washington, Seattle, WA USA; 7 Howard Hughes Medical Institute, University of Washington, Seattle, WA USA

**Keywords:** Bacteria, Imaging

## Abstract

Advances in microscopy hold great promise for allowing quantitative and precise measurement of morphological and molecular phenomena at the single-cell level in bacteria; however, the potential of this approach is ultimately limited by the availability of methods to faithfully segment cells independent of their morphological or optical characteristics. Here, we present Omnipose, a deep neural network image-segmentation algorithm. Unique network outputs such as the gradient of the distance field allow Omnipose to accurately segment cells on which current algorithms, including its predecessor, Cellpose, produce errors. We show that Omnipose achieves unprecedented segmentation performance on mixed bacterial cultures, antibiotic-treated cells and cells of elongated or branched morphology. Furthermore, the benefits of Omnipose extend to non-bacterial subjects, varied imaging modalities and three-dimensional objects. Finally, we demonstrate the utility of Omnipose in the characterization of extreme morphological phenotypes that arise during interbacterial antagonism. Our results distinguish Omnipose as a powerful tool for characterizing diverse and arbitrarily shaped cell types from imaging data.

## Abstract (web summary)

Omnipose is a deep neural network algorithm for image segmentation that improves upon existing approaches by solving the challenging problem of accurately segmenting morphologically diverse cells from images acquired with any modality.

## Main

Although light microscopy is a valuable tool for characterizing cellular and subcellular structures and dynamics, quantitative analysis of microscopy images remains a persistent challenge^1^. This is especially pertinent to the study of bacteria, many of which have dimensions in the range of visible wavelengths. Thus, their cell body is composed of a small number of pixels (for example, ~100–300 px^2^ for *Escherichia* *coli* in typical experiments). At this scale, accurate subcellular localization requires defining the cell boundary with single-pixel precision. The process of defining boundaries within images is termed segmentation, and this is a critical first step in image analysis pipelines^2,3^.

In addition to their small size, bacteria adopt a wide range of morphologies. Although many commonly studied bacteria are well approximated by rods or spheres, there is growing interest in bacteria with more elaborate shapes^4^. Some examples include Streptomycetales, which form long filamentous and branched structures^5^, and Caulobacterales, which possess extended appendages distinct from their cytoplasm^6^. Furthermore, microfluidic devices are allowing researchers to capture the responses of bacteria to assorted treatments such as antibiotics, which often result in highly irregular morphologies^7^. Whether native or induced, atypical cell morphologies present a distinct problem at the cell-segmentation phase of image analysis^8,9^. This is compounded when such cells are present alongside those adopting other morphologies, as is the case in many natural samples of interest^10^. Although some algorithms can be trained to segment objects imaged using assorted modalities, to date there is no generalizable solution for segmenting bacterial cells of assorted size and shape^1^.

Cell segmentation is a complex problem that extends beyond microbiological research; thus, many solutions are currently available in image-analysis programs^8,9,11–27^. Most of these solutions use traditional image-processing techniques such as intensity thresholding to segment isolated cells; however, this approach performs poorly on cells in close contact and it requires image-by-image tuning to optimize parameters. SuperSegger was developed to address thresholding issues specifically in bacterial phase-contrast images^13^. This program utilizes both traditional image filtering techniques and a shallow neural network to correct for errors that thresholding and watershed segmentation tend to produce.

Deep neural networks (DNNs) are now widely recognized as superior tools for cell segmentation^28^. Unlike traditional image processing, machine-learning approaches such as DNNs require training on a ground-truth dataset of cells and corresponding labels. Trained DNNs are thus limited in applicability to images that are representative of those in the training dataset. Early DNN approaches were based on the Mask R-CNN architecture^24^, whereas more recent algorithms such as StarDist, Cellpose and MiSiC are based on the U-net architecture^12,15,26^. Pachitariu and colleagues showed that Cellpose outperforms Mask R-CNN and StarDist on a variety of cell types and cell-like objects, distinguishing it as a general solution for cell segmentation^12^. Notably, the representation of bacteria in their study was limited. MiSiC was developed as a general DNN-based solution for bacterial segmentation; however, the authors of MiSiC did not provide comparisons to other DNN algorithms^15^. Here, we evaluated the performance of state-of-the-art cell segmentation algorithms on a diverse collection of bacterial cells. Our findings motivated the design of a new algorithm, Omnipose, which substantially outperforms all segmentation algorithms tested across a wide range of bacterial cell sizes, morphologies, and optical characteristics. We have made Omnipose and all associated data available to researchers and we anticipate that our model, without retraining, can be applied to diverse bacteriological systems. Furthermore, following the incorporation of additional ground-truth data, Omnipose could serve as a platform for segmenting various eukaryotic cells and extended, anisotropic objects more broadly.

## Results

### Evaluation of bacterial cell segmentation algorithms

Numerous image segmentation algorithms have been developed and the performance of many of these on bacterial cells is documented^1^. These broadly fall into three categories: (1) traditional image-processing approaches (for example, thresholding and watershed); (2) traditional/machine-learning hybrid approaches; and (3) DNN approaches. Given the goal of developing software with the capacity to recognize bacteria universally, we sought to identify strongly performing algorithms for further development. An unbiased, quantitative comparison of cell segmentation algorithms on bacterial cells has not been performed; thus, we selected one or more representatives from each category for our analysis: Morphometrics^23^ (1); SuperSegger^13^ (2); and Mask R-CNN^27^, StarDist^26^, MiSiC^15^, and Cellpose^12^ (3). A detailed review of these choices is in the Methods.

**Extended Data Fig. 1:**
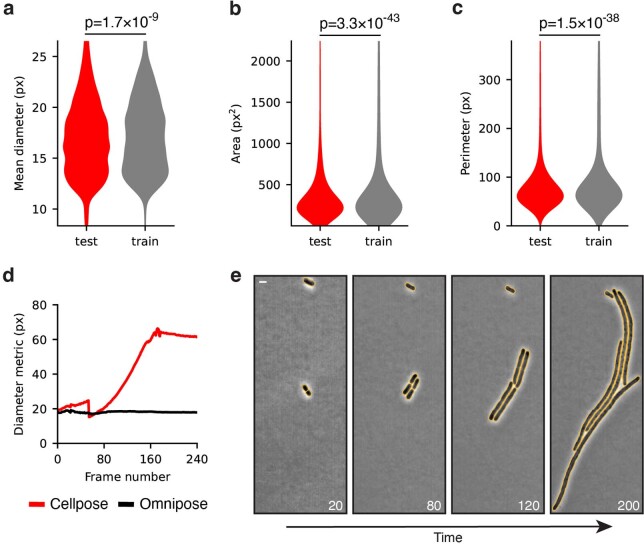
Size and morphology metrics are indistinguishable between cell populations included in training and test datasets. (**a**) Mean cell diameter, (**b**) cell area, and (**c**) cell perimeter calculated for our bacterial phase-contrast ground-truth dataset. P-values are displayed for the two-sided KS test. n = 47,000 (27,500 for training, 19,500 for testing). **(d**). Comparison of diameter metrics of a timelapse of elongated cell growth. The Cellpose diameter metric is the diameter of a circle with equivalent area. Omnipose diameter metric is proportional to the mean of the distance transform (see Methods). (**e**) Bacteria displayed are a single population analyzed of *A. baylyi* transformed with a Δ*ftsN::kan* PCR fragment. Yellow lines indicate cell label boundaries. Scale bar is 1 μm.

For training and benchmarking of these algorithms, we acquired micrographs of assorted bacterial species representing diverse morphologies and optical characteristics. Many studies of bacteria involve mutations or treatments that cause extreme morphologies. To capture this additional diversity, we included wild-type and mutant bacteria grown in the presence of two β-lactam antibiotics, cephalexin and aztreonam, and A22, which targets MreB^29^. Finally, based on our interest in microbial communities, we acquired images of mixtures of bacterial species which display distinct morphologies and optical characteristics. In total, we collected 4,833 images constituting approximately 700,900 individual cells deriving from 14 species (Supplementary Table 1). Next, we developed a streamlined approach for manual cell annotation and applied it to these images (Methods), yielding 47,000 representative annotated cells that serve as our ground-truth dataset (bact_phase). We divided this data into a 27,500-cell training set and a 19,500-cell benchmarking set. Relevant cellular metrics (area, perimeter and mean diameter) did not differ substantially between the groups, confirming that the benchmarking set faithfully represents the training set (Extended Data Fig. 1).

**Fig. 1:**
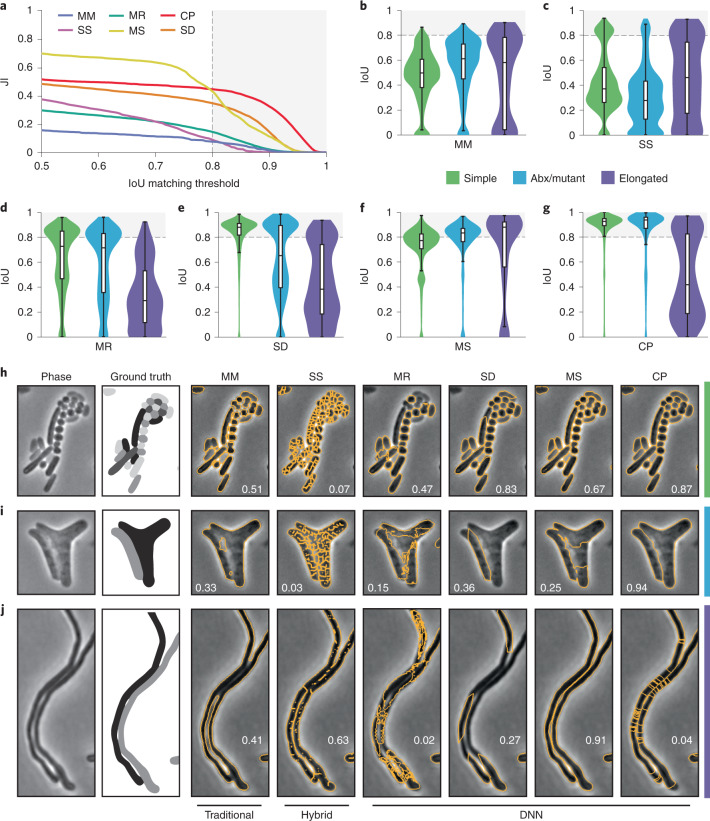
Quantitative comparison of segmentation methods distinguishes Cellpose as a high-performing algorithm. **a–g**, Comparison of segmentation algorithm performance on our bact_phase test dataset (*n* = 19,538 cells). Overall performance measured by JI (**a**). MM, Morphometrics; MR, Mask R-CNN; CP, Cellpose; SS, SuperSegger; MS, MiSiC; SD, StarDist. The JI was calculated at the image level and values averaged across the dataset are displayed. Algorithm performance was partitioned by cell type (simple, *n* = 12,869; Abx/mutant, *n* = 6,138; elongated, *n* = 531) (**b–g**). Images were sorted into types as defined in Supplementary Table 1. Abx, antibiotic. Boxes are centered on medians from Q1 to Q3, whiskers from Q1 − 1.5 IQR to Q3 + 1.5 IQR. IQR, interquartile range Q3–Q1. **h–j**, Representative micrographs of cell type partitions analyzed in **b**–**g**, indicated by vertical bars on right. Ground-truth masks and predicted mask outlines generated by the indicated algorithm are displayed. Mean matched IoU values for cells shown are displayed within each micrograph. Bacteria displayed are *Vibrio* *cholerae*, *Pseudomonas* *aeruginosa*, *Bacillus* *subtilis*, and *Staphylococcus* *aureus* (**h**), aztreonam-treated *E.* *coli* CS703-1 (**i**), and *Streptomyces* *pristinaespiralis* (**j**). All images are scaled equivalently. Scale bar, 1 μm.

**Extended Data Fig. 2:**
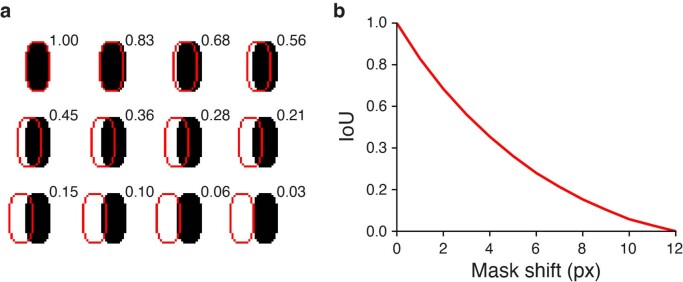
The relationship between IoU and segmentation accuracy. (**a**) Illustration of 0–12 pixel displacement of cell mask (red outline) and corresponding IoU values using a synthetic cell of typical bacterial size and resolution (solid black). (**b**) Quantification of the impact of mask shift on IoU values, determined using the synthetic cell shown in (a).

To facilitate direct comparison of the algorithms, we first optimized their performance against our data. For the DNN approaches, each algorithm was trained on our dataset using developer-recommended parameters. Morphometrics and SuperSegger cannot be automatically optimized using ground-truth data; therefore, we manually identified settings that optimized the performance of these algorithms against our dataset (Methods). As a quantitative measure for algorithm performance, we compared their average Jaccard index (JI) as a function of intersection over union (IoU) threshold (Fig. 1a)^30,31^. IoU values lie between zero and one, with values greater than 0.8 marking the point at which masks become indistinguishable from ground truth by the expert human eye (Extended Data Fig. 2)^30^. This analysis showed that DNN-based approaches outperform other algorithms; however, substantial differences in performance within the DNN group were observed. Cellpose and StarDist outperform Mask R-CNN and MiSiC at high IoU thresholds. The performance of all algorithms varied greatly across images in the bact_phase dataset, with much of this variability delineated by cell type and morphology categories (Fig. 1b–g). Whereas all other algorithms exhibited visible segmentation errors in two of the three cell categories we defined, errors by Cellpose were only apparent in elongated cells (Fig. 1h–j).

### Motivation for a new DNN-based segmentation algorithm

Our comparison revealed that Cellpose offers superior performance relative to the other segmentation algorithms we analyzed, and for this reason we selected this algorithm for further development. Notably, even at the high performance levels of Cellpose, only 81% of predictions on our benchmarking dataset are above 0.8 IoU. This limits the feasibility of highly quantitative studies such as those involving subcellular protein localization or cell–cell interactions.

**Extended Data Fig. 3:**
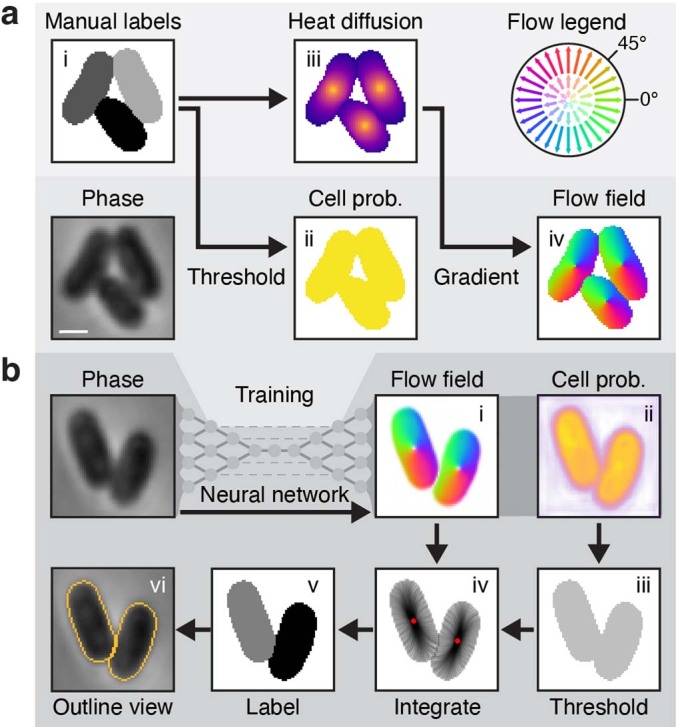
Details of the Cellpose algorithm. (**a**) Stages of the Cellpose training pipeline. Ground truth masks (*i*) are converted to cell probability (*ii*) by binary thresholding and a heat distribution (*iii*) by simulated diffusion from the median pixel coordinate. The flow field (*iv*) is defined by the normalized gradient of (*iii*). Color-magnitude representations of this vector field follow the flow legend diagram. The phase, cell probability and flow fields are used to train the network. (**b**) Stages of the cellpose prediction pipeline. Phase images are processed by the trained cellpose network into the intermediate flow field and cell probability outputs (*i-ii*). A binary threshold is applied to the probability to identify cell pixels (*iii*). Pixels are Euler-integrated under the flow field until they converge at common points. Boundary pixel trajectories are depicted in *iv*. Each pixel is assigned a unique label corresponding to the center to which it converged (*v*). This segmentation result is commonly depicted in an outline view (*vi*). Bacteria shown are *Escherichia coli*. Scale bar is 1 μm.

Cellpose utilizes a two-step process. Its neural network first transforms an input image into several intermediate outputs, including a scalar probability field for identifying cell pixels in the next step (Extended Data Fig. 3a(i)–(iii))^12^. Cellpose is unique among DNN algorithms by the addition of a vector field output (the flow field), which is defined by the normalized gradient of a heat distribution from the median cell pixel coordinate (Extended Data Fig. 3a(iv),(v)). In the second step, this vector field directs pixels toward a global cell center via Euler integration, thereby segmenting cells based on the points at which pixels coalesce (Extended Data Fig. 3b). In contrast to other algorithms, this approach for reconstructing cell masks is size- and morphology-independent, insofar as the cell center can be correctly defined.

**Extended Data Fig. 4:**
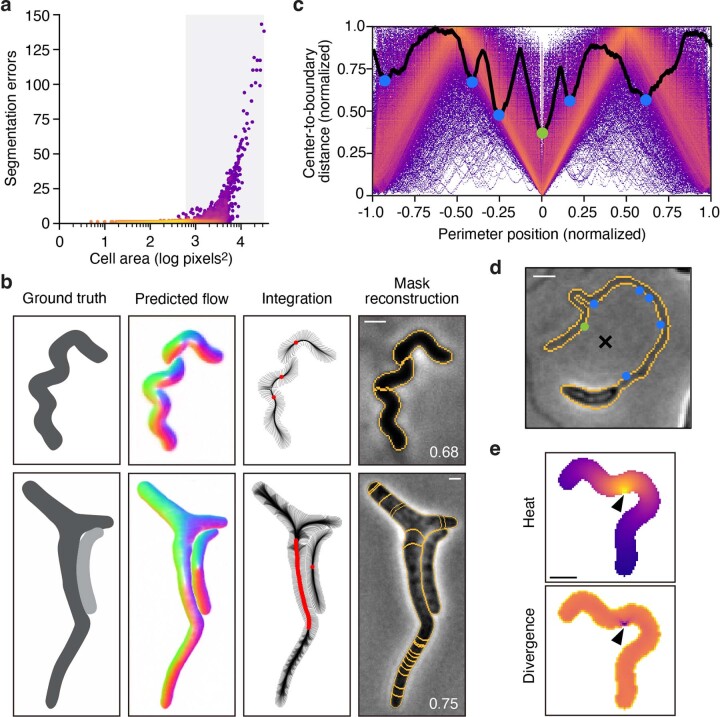
Cellpose over-segments extended, anisotropic cells. (**a**) Single-cell analysis of Cellpose segmentation error as a function of cell area. Color represents density on a log scale. Gray box represents the top quartile of cell areas (n = 19,570). (**b**) Example images representative of 1,128 cells with segmentation errors in the top area quartile (n = 4,887). Corresponding boundary pixel trajectories are shown in black and final pixel locations in red. Predicted mask overlays are shown with mean matched IoU values. Cellpose model bact_phase_cp used in (a,b). (**c**) Analysis of stochastic center-to-boundary distances in our ground-truth dataset. Distance from the center (median pixel coordinate) to each boundary pixel is normalized to a maximum of 1. Position along the boundary is normalized from −1 to 1 and centered on the point closest to the median pixel. Center-to-boundary for the cell in (d) is highlighted in black. (**d**) Representative cell with median coordinate outside the cell body (black X). Cellpose projects this point to the global minima of this function (green dot), but several other local minima exist (blue dots). (**e**) The heat distribution resulting from a projected cell center (black arrow). The normalized gradient corresponds to the divergence shown. (d-e) represent n = 617 cells with projected centers in the training dataset. Bacteria displayed are (a,e) *Helicobacter pylori*, (b) *Escherichia coli* CS703-1, both treated with aztreonam and (d) *Caulobacter crescentus* grown in HIGG media. Scale bars, 1 μm.

To understand the mechanisms behind Cellpose segmentation errors, we evaluated its performance as a function of cell size on our bact_phase dataset. We compared cell area against the number of segmentation errors, calculated as the number of redundant or missing masks corresponding to each ground-truth cell mask. This revealed a strong correlation between cell size and segmentation errors, with the top quartile of cells accounting for 83% of all errors (Extended Data Fig. 4a). To understand the source of these errors, we inspected the flow field output of many poorly segmented cells across a variety of species and growth conditions. This showed that elongated cells, an important morphology often seen in both wild-type and mutant bacterial populations, are particularly susceptible to over-segmentation (Extended Data Fig. 4b). We attribute this to the multiple sinks apparent in the corresponding flow fields. In the Cellpose mask reconstruction algorithm, pixels belonging to these cells are guided into multiple centers per cell, fragmenting the cell into many separate masks.

**Extended Data Fig. 5:**
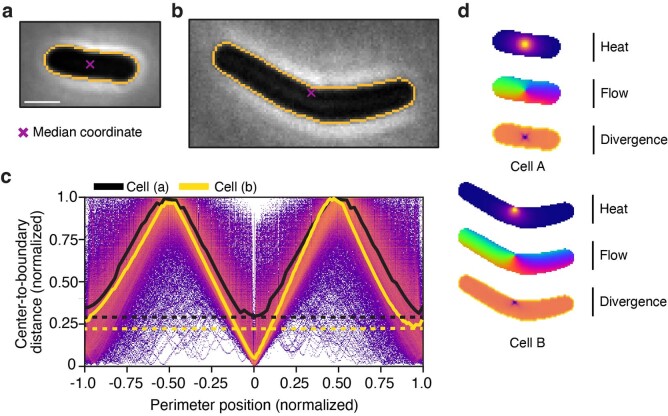
Median coordinates used to generate Cellpose ground-truth flow fields are asymmetrically localized for some bacterial cell morphologies. (**a**) Rod-shaped *E. coli* with symmetric median coordinate. Symmetry of the center is reflected in A by equal high and low points corresponding to the extremal points along the long and short axes of the cell. (**b**) Curved *B. subtilis* with median coordinate asymmetrically close to the cell boundary. This asymmetry is reflected in A by a secondary minimum above the global minimum corresponding to the diametrically opposing point along the short axis of the cell. (**c**) Center-to-boundary distance highlighted for cells A (black) and B (yellow) with non-projected median coordinates. Dashed lines indicate the larger of the two minima along the medial axis. (**d**) Flow fields generated by Cellpose for cells A and B. Scale bar is 1 μm. Images scaled equivalently.

We hypothesized that the defect in Cellpose flow field output is a consequence of two distinct flow field types arising from our training dataset: those where the median pixel coordinate, or ‘center’, lies within the cell (97.8%) and those where it lies outside the cell (2.2%). In the latter, Cellpose projects the center point to the nearest boundary pixel, ultimately leading to points of negative divergence on cell peripheries that are chaotically distributed (Extended Data Fig. 4c–e). On the contrary, non-projected centers maintain a uniform field magnitude along the entire boundary and adhere to the global symmetries of the cell (Extended Data Fig. 5a,d). A similar issue is also encountered in cells with centers that fall close to but not outside of the boundary (Extended Data Fig. 5b–d). Cells with a center point closer than 0.3 times the mean cell diameter (a factor of 0.2 off-center) to the boundary account for an additional 9.6% of our training dataset. Neural networks can be exquisitely sensitive to the outliers in their training data^32^; therefore, we suspect that this small fraction of corrupt flow fields has a disproportionate impact on the performance of Cellpose.

### Development of a high-precision U-net segmentation algorithm

We sought to develop a segmentation algorithm that operates independently of cell center identification. The algorithm we developed is based on the framework of Cellpose, which can be divided into five key components: file handling, neural network architecture, training objective functions, network predictions, and mask reconstruction. We made improvements to each of these components; however, the major innovations in our algorithm, which we named Omnipose, pertain to network predictions and mask reconstruction.

**Extended Data Fig. 6:**
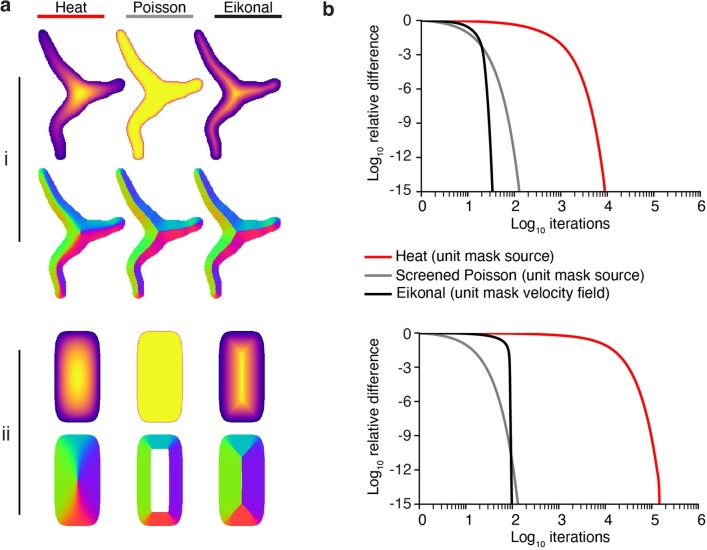
The Eikonal equation provides a fast and accurate flow field calculation for diverse cell morphologies and sizes. (**a**) Partial differential equation solutions (top rows) and corresponding flow fields (bottoms rows) calculated for two examples cells (i, ii) using a relaxation algorithm for the heat, Poisson and Eikonal equations. Cell (i) is drawn from our dataset (mean diameter 37px) and cell (ii) is a synthetic rod-shaped cell (mean diameter 192px). (**b**) Convergence measured by the average difference at each iteration (maximum normalized to 1) for cells (*i,ii*).

Unlike the cell probability and center-seeking flow field upon which Cellpose is constructed, we built Omnipose on three distinct network outputs: a cell boundary probability map, the distance field, and a flow field defined by the gradient of the distance field. The distance field (or distance transform) describes the distance at any point $\vec x$ in a bounded region Ω to the closest point on the boundary ∂Ω. Notably, this widely utilized construct is one of the intermediate outputs of StarDist^32^. Whereas StarDist uses a distance field prediction to seed and assemble star-convex polygons, Omnipose implements the distance field as a replacement to the cell probability output of Cellpose. The use of a distance field has several advantages. First, the distance field is more structured than a binary probability map and offers higher fidelity thresholding to seed cell masks. Second, the distance field is defined by the eikonal equation $| {\vec \nabla {{\Phi }}\left( {\vec x} \right)} | = 1$ and so its gradient (the flow field of Omnipose) has unit magnitude throughout the bounded region for which it is calculated. This leads to faster convergence and better numerical stability when compared to alternative solutions producing similar fields (for example, screened Poisson) (Methods and Extended Data Fig. 6a). Third, the distance field is independent of morphology and topology, meaning that it is applicable to all cell shapes and sizes. Last, the resulting flow field points uniformly from cell boundaries toward the local cell center, coinciding with the medial axis (skeleton) that is defined by the stationary points of the distance field (Extended Data Fig. 6b). This critical feature allows pixels to remain spatially clustered after Euler integration, solving the problem of over-segmentation seen in Cellpose.

**Fig. 2:**
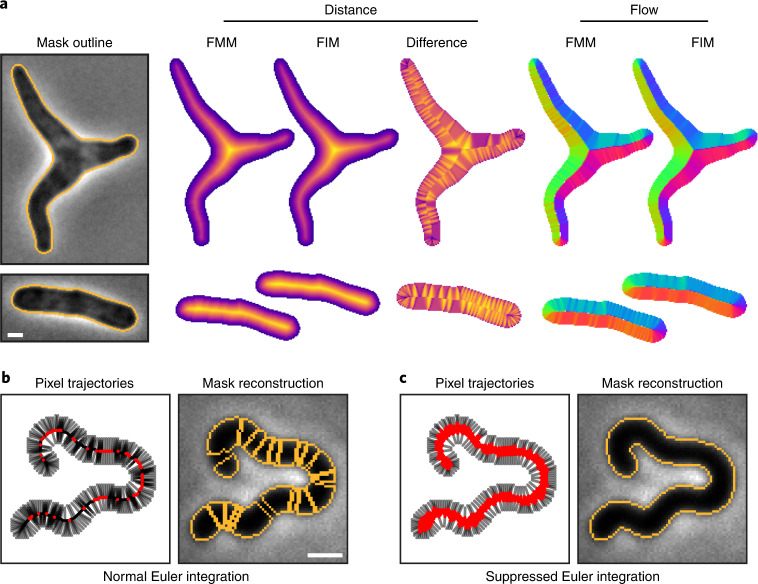
Core innovations of Omnipose. **a**, Comparison of distance field algorithms and corresponding flow fields on ground-truth masks. FMM produces ridges in the distance field resulting from pixelation on the cell mask boundary. Our smooth FIM algorithm minimizes these features. The difference image (FIM − FMM) highlights artifacts in the FMM method. Flow fields are calculated as the normalized gradient of the distance field. Boundary pixelation affects the FMM flow field deep into the cell, regardless of cell size. **b**,**c**, Comparison of mask reconstruction algorithms on a smooth flow field. Boundary pixel trajectories and resulting mask outlines from standard Euler integration (**b**). Trajectories and mask outlines under suppressed Euler integration (**c**). Red dots indicate the final positions of all cell pixels, not only the boundary pixels for which trajectories are displayed. Bacteria displayed are *E.* *coli* CS703-1 (**a**) and *H.* *pylori* (**b**,**c**) both treated with aztreonam. Scale bars, 1 μm. Images are representative of 1,299 *E.* *coli* and 701 *H.* *pylori* cells in the total ground-truth dataset, respectively.

One challenge to implementing a distance-field-based approach is that traditional distance field algorithms such as fast marching method (FMM) are sensitive to boundary pixelation^33^, causing artifacts in the flow field that extend deep into the cell. These artifacts are sensitive to pixel-scale changes at the cell perimeter, which we reasoned would interfere with the training process. To solve this problem, we developed an alternative approach based on fast iterative method (FIM) that produces smooth distance fields for arbitrary cell shapes and sizes (Fig. 2a, Extended Data Fig. 6 and Methods)^34^. The corresponding flow field is relatively insensitive to boundary features at points removed from the cell boundary, a critical property for robust and generalized prediction by the Cellpose network.

The use of the distance field additionally required a unique solution for mask reconstruction. Whereas the pixels in a center-seeking field converge on a point, standard Euler integration under our distance-derived field tends to cluster pixels into multiple thin fragments along the skeleton, causing over-segmentation (Fig. 2b). We solved this with a suppression factor of (*t* + 1)^−1^ in each time step of the Euler integration (Fig. 2c). This reduces the movement of each pixel after the first step *t* = 0, facilitating initial cell separation while preventing pixels from clustering into a fragmented skeleton formation. The wider point distribution resulting from our suppression factor allows pixels to remain connected, thereby generating a single mask for each cell in conjunction with a standard automated point clustering algorithm (for example, DBSCAN)^35^.

### Omnipose demonstrates unprecedented segmentation accuracy

**Fig. 3:**
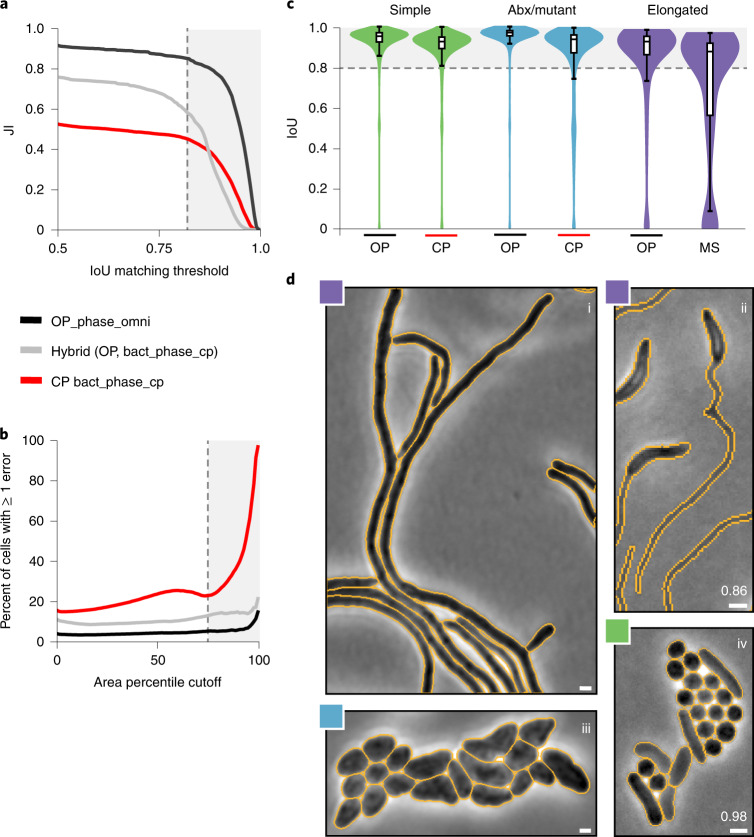
Omnipose substantially outperforms Cellpose on elongated cells. **a**, Overall performance of Omnipose (OP) (bact_phase_omni) and Cellpose (CP) (bact_phase_cp) measured by JI. The hybrid method (gray) uses the original center-seeking flow output of bact_phase_cp and the mask reconstruction of Omnipose. Gray box represents IoU ≥ 0.8. *n* = 19,570 cells in the test set. **b**, Quantification of segmentation performance by cell size. The percent of cells with at least one segmentation error is computed for cells in each area percentile group from 1 to 100. Gray box represents the top quartile. **c**, Omnipose IoU distribution on the bact_phase dataset compared to the next highest performing algorithm in each of three cell categories (simple, *n* = 12,869; Abx/mutant, *n* = 6,138; and elongated, *n* = 531). Boxes centered on medians from Q1 to Q3, whiskers from Q1 − 1.5 IQR to Q3 + 1.5 IQR. **d**, Example micrographs and Omnipose segmentation. Mean matched IoU values shown. Bacteria displayed are *Streptomyces* *pristinaespiralis* (i), *Caulobacter* *crescentus* grown in HIGG medium (ii), *Shigella* *flexneri* treated with A22 (iii) and a mix *of Pseudomonas* *aeruginosa, Staphylococcus* *aureus, V.* *cholerae*, and *Bacillus* *subtilis* (iv). HIGG, Hutner base–imidazole-buffered glucose–glutamate. Scale bars, 1 μm.

To benchmark the performance of Omnipose, we trained a model (bact_phase_omni) on our bact_phase dataset. Remarkably, across the IoU threshold range 0.5–1.0, the accuracy of Omnipose substantially exceeds that of Cellpose using a corresponding model (bact_phase_cp) (Fig. 3a). This difference in performance between the algorithms is particularly pronounced within the high IoU range 0.8–1.0. Bacteria are 0.5–5 μm in scale and are typically imaged with a calibrated pixel size of about 0.1 μm, resulting in cells and cell labels that are 5–50 pixels across^36^. Quantitative measurements at this scale require pixel-level accuracy, corresponding to IoU values above 0.8 (Extended Data Fig. 2). Thus, Omnipose is uniquely suited for the microscopic analysis of bacterial cells.

To dissect the contributions of the individual Omnipose innovations to the overall performance of the algorithm, we isolated the mask reconstruction component of Omnipose and applied it to the Cellpose network output. This augmentation of Cellpose modestly improved its performance across all IoU thresholds (Fig. 3a). Based on this, we attribute the remaining gains in performance by Omnipose to its unique network outputs (boundary, distance, and flow) and to our improvements to the Cellpose training framework. The latter includes numerous custom loss functions, the use of an alternative optimizer (RAdam), and image augmentations (Methods).

**Extended Data Fig. 7:**
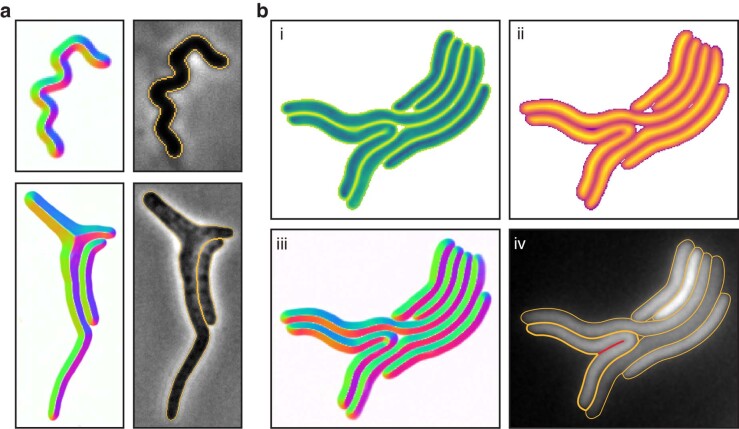
Omnipose output on diverse morphologies. **(a)** Omnipose flow and segmentation corresponding to the cells of Extended Data Fig. 4b. **(b)** (*i-iii*) Boundary, distance and flow output using bact_fluor_omni model on *S. flexneri* treated with cephalexin. (*iv*) Overlaid mask outlines. The cell in bold yellow is missing the self-contact boundary in red.

Our analyses illuminated critical flaws in previous DNN-based approaches for the segmentation of elongated cells, effectively preventing these algorithms from generalizable application to bacteria (Fig. 1). To determine whether Omnipose overcomes this limitation, we evaluated its performance as a function of cell area. Cell area serves as a convenient proxy for cell length in our dataset, which is composed of both branched and unbranched elongated cells. While the Cellpose cell error rate remains above 15% across all cells, the Omnipose error rate does not exceed 5% before the 90th percentile of cell area is reached (Fig. 3b). Thus, Omnipose performance is independent of cell size and shape, including those cells with complex, extended morphologies (Fig. 3c,d and Extended Data Fig. 7a).

### Omnipose is a multifunctional segmentation tool

**Fig. 4:**
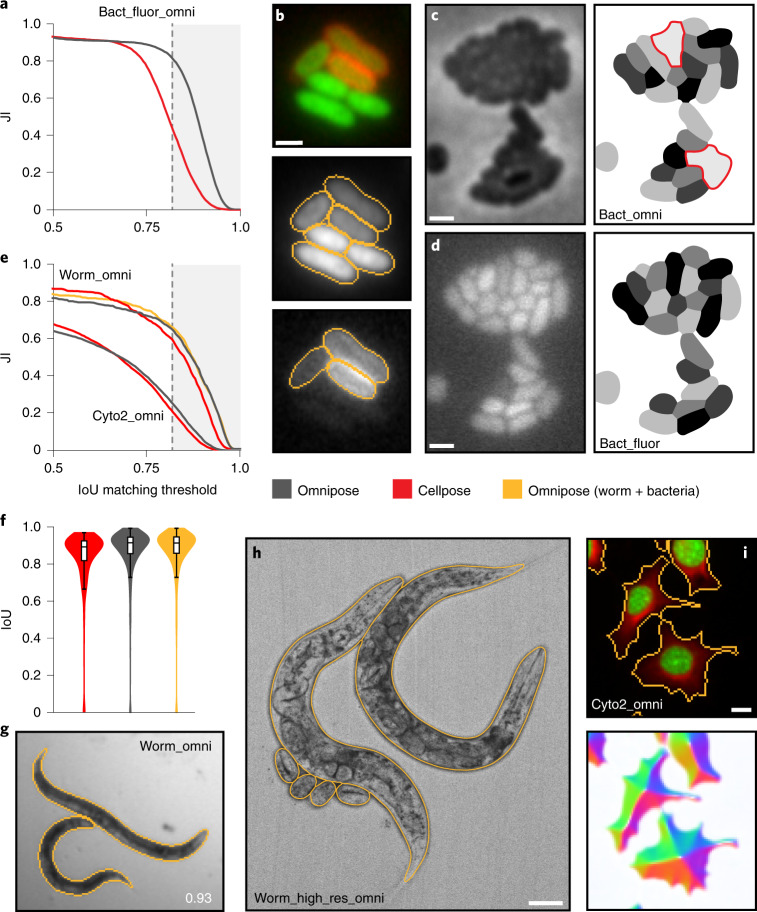
Omnipose models trained and evaluated on assorted imaging modalities and subjects. Relevant Omnipose model names are provided (also Supplementary Table 2). **a**, Performance of bacterial fluorescence Omnipose (bact_fluor_omni) and Cellpose (bact_fluor_cp) models. Test set consists of *n* = 14,587 cells. **b**, Fluorescence micrograph (top) and corresponding bact_fluor_omni segmentation results of *B.* *thailandensis* expressing cytoplasmic green fluorescent protein (GFP) (segmentation, middle) or outer-membrane localized mCherry (segmentation, bottom). **c**,**d**, *Francisella* *tularensis* subsp *novicida* segmentation using bact_phase_omni (**c**) or bact_fluor_omni (**d**) Omnipose models. Two notable bact_phase_omni segmentation errors are highlighted in red. Scale bars, 1 μm. **e**, Performance of Omnipose models trained on cyto2 (cyto2_omni, *n* = 10,232) and *C.* *elegans* (worm_omni) datasets versus corresponding Cellpose models. Results for Omnipose and Cellpose trained on either *C.* *elegans* alone (gray, red) or Omnipose on *C.* *elegans* and bacterial data (yellow) are shown. Cyto2 test dataset, *n* = 10,232 cells. *C.* *elegans* test dataset, *n* = 1,264 worms. **f**, IoU distribution for the masks predicted by each method on our *C.* *elegans* test dataset (*n* = 1,264). Boxes centered on medians from Q1 to Q3, whiskers from Q1 − 1.5 IQR to Q3 + 1.5 IQR. **g**, Example segmentation of *C.* *elegans* in the BBBC010 dataset used in **e**,**f**. IoU score shown. Scale bar, unavailable. **h**, Example segmentation of high-resolution *C.* *elegans* (minimum projection brightfield). Scale bar, 50 μm. **i**, Example multi-channel Omnipose segmentation in the cyto2 dataset. Scale bar, 3 μm.

We have shown that the features of Omnipose improve bacterial phase-contrast segmentation performance beyond that of Cellpose. Like other DNN-based algorithms, Omnipose can also segment images acquired using different modalities and composed of alternative subjects when trained on a representative dataset. To evaluate Omnipose on images acquired using a modality distinct from phase contrast, we curated a dataset containing cytosol and membrane fluorescence in 33,200 bacterial cells (bact_fluor). In brief, this was achieved by applying the labels of cells with fluorescence signal in our phase-contrast ground-truth images to their corresponding fluorescence channels (Methods). As expected, we found that the enhanced performance of Omnipose (bact_fluor_omni) relative to Cellpose (bact_fluor_cp) on morphologically diverse cells translates to fluorescence images (Fig. 4a–d and Extended Data Fig. 7b).

**Extended Data Fig. 8:**
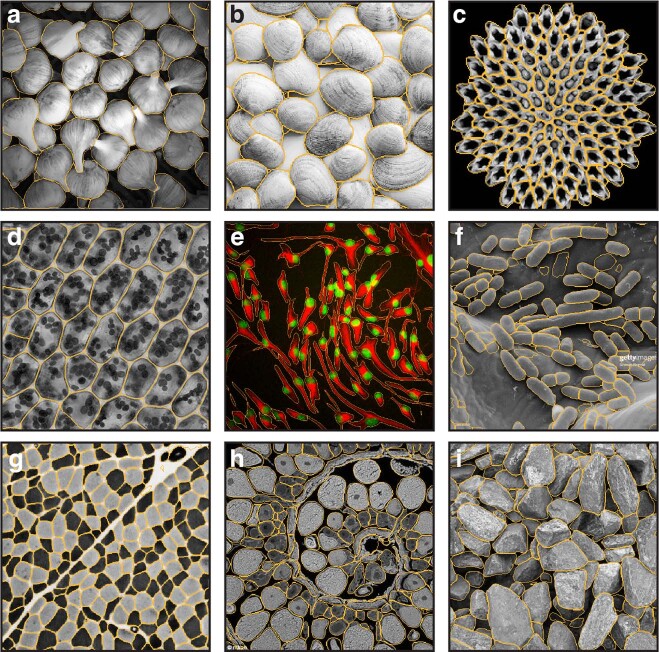
Omnipose accurately segments diverse images from the cyto2 test dataset. **(a-i)** Selection of images from the cyto2 test dataset with superimposed outlines representing Omnipose cyto2_omni model segmentation. Subjects are not defined, as the cyto2 dataset lacks metadata and is comprised of anonymized contributions.

We next sought to investigate the potential utility of Omnipose in the segmentation of non-bacterial subjects. The nematode *Caenorhabditis* *elegans* is a widely studied model organism with an overall morphology similar to elongated bacteria^37^. At just 1 mm in length, *C.* *elegans* phenotypes are often analyzed by timelapse microscopy; therefore, there is a need for segmentation methods that enable accurate tracking^38^. We obtained, annotated, and trained Omnipose on two publicly available, low-resolution microscopy datasets composed of *C.* *elegans* images: timelapse frames from the Open Worm Movement database^39^ and frames containing fields of assorted live or dead *C.* *elegans* from the BBBC010 dataset^40^. Many images in this combined worm dataset contain debris and are of heterogenous quality, yet 83% of masks predicted by Omnipose match or exceed the 0.8 IoU threshold (Fig. 4f). At the low resolution of our combined *C.* *elegans* dataset, the worms approximate bacteria in shape and diameter (in pixels). Indeed, we found that a generalist model trained on both our extensive bact_phase dataset and our worm dataset (worm_bact_omni) exhibited equivalent or higher performance relative to the specialist worm model (worm_omni). We also trained an Omnipose model using a custom collection of high-resolution *C.* *elegans* images (worm_high_res) and found that the algorithm can successfully segment these images despite the complex internal structure (Fig. 4h). Cyto2 is a large collection of images and corresponding ground-truth annotations submitted by Cellpose users that expands upon the original cyto dataset developed to evaluate Cellpose^12,30^. This dataset includes many non-cell images (for example, rocks, onions) as well as multi-channel fluorescence images (for example, cytosol and nuclear stains in mammalian cells) (Fig. 4i and Extended Data Fig. 8). We found that Omnipose offers a modest improvement in performance relative to Cellpose on the cyto2 dataset (Fig. 4e) and this was achieved without compromising the segmentation rate (~1 image per second).

**Fig. 5:**
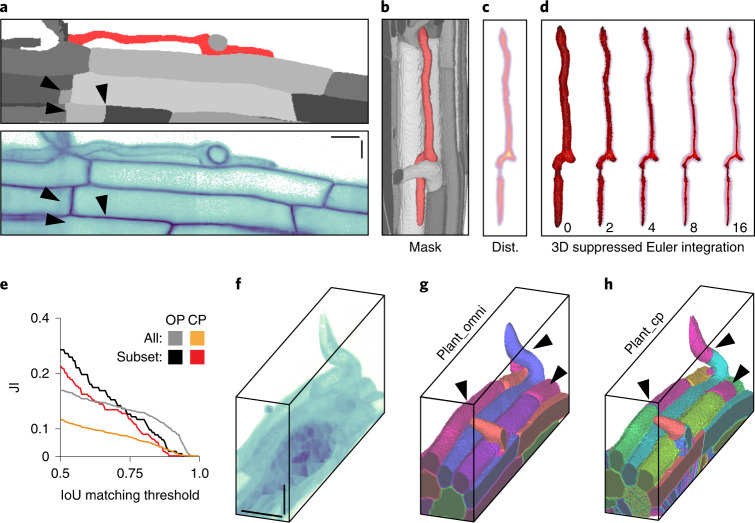
Omnipose can be applied to 3D datasets. **a**, Volume slice in the *A.* *thaliana* 3D training set. Black arrows denote over-segmentation in the ground truth labels. Red corresponds to the cell in **b**–**d**. **b**, Selected cell in context of ground-truth data. **c**, Ground-truth distance field for selected cell in **b**. **d**, Steps of the suppressed Euler integration for selected cell in **b**. Boundary pixels shown, colored by overall displacement (dark to light red). **e**, Performance of Omnipose (OP, plant_omni) and Cellpose (CP, plant_cp) on the full test dataset (*n* = 604) and on a subset without excluded regions (Methods) depicted in **f**–**h** (*n* = 73). **f–h**, Ground-truth masks (**f**) and segmentation results of *A.* *thaliana* using Omnipose (**g**) and Cellpose (**h**). Scale bars, 20 μM.

**Extended Data Fig. 9:**
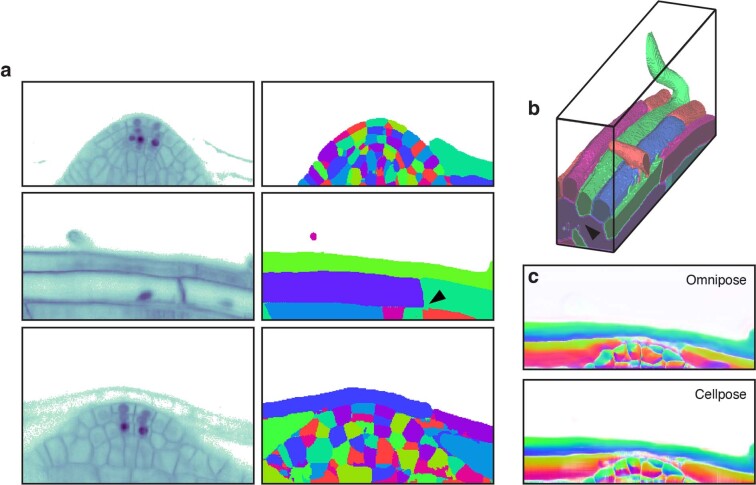
Errors in the three-dimensional *A. thaliana* ground-truth dataset impact training and performance metrics of Ominpose and Cellpose. **(a)** Slices of ground truth training set volumes. Images show errant pixels and apparent flaws in ground truth masks. Black arrow indicates one instance of a joined mask. **(b)** Ground truth masks for the test volume in Fig. 5e-h. Two cells are joined under one label (black arrow). **(c)** Slices of the predicted Omnipose and Cellpose flow fields through the middle of the volume in (b).

Two-dimensional (2D) imaging allows the characterization of cells within constrained environments, yet many phenomena of interest can only be studied in natural, three-dimensional (3D) contexts. We modified each explicitly 2D component of the network architecture, outputs and mask reconstruction elements of Omnipose to enable direct segmentation of 3D (or higher order) data. This contrasts with the solution adopted for Cellpose (Cellpose3D), which approximates a 3D field by combining the 2D flow components predicted on orthogonal 2D volume slices^12^. We compared Cellpose3D to Omnipose on a publicly available *Arabidopsis* *thaliana* lateral root primordia dataset acquired using confocal microscopy^22^. An Omnipose model was trained on six volumes representing 931 cells in total (plant_omni), whereas Cellpose was trained on 3,070 slices of these volumes (plant_cp) (Methods). Consistent with our results in 2D, Omnipose provided more accurate segmentation results than Cellpose, particularly among elongated cells in the dataset (Fig. 5). We note that the absolute scores for both algorithms are low and attribute this in part to inaccurate ground-truth masks (Extended Data Fig. 9a,b). Indeed, in certain instances, Omnipose-predicted masks seem to be more accurate than the ground truth (Fig. 5b and Extended Data Fig. 9b). Notably, the 3D field reconstruction made in Cellpose from 2D plant_cp slice predictions recapitulates much of the direct 3D field predictions of plant_omni (Extended Data Fig. 9c). We reason that this is because the local 2D cell slice ‘centers’ of the Cellpose flow field can be coincident with the global 3D cell ‘skeletons’ of the Omnipose flow field. Taken together with our results on diverse cell types and fluorescence images, we conclude that Omnipose maintains the multi-modal segmentation capabilities of Cellpose while adding the ability to perform accurate segmentation of a substantially broader range of cellular morphologies present within 2D and 3D data.

### Omnipose permits sensitive detection of cell intoxication

Our laboratory recently described an interbacterial type VI secretion system-delivered toxin produced by *Serratia* *proteamaculans*, Tre1 (ref. ^41^). We showed that this toxin acts by ADP-ribosylating the essential cell division factor FtsZ; however, we were unable to robustly evaluate the consequences of Tre1 intoxication on target cell morphology due to segmentation challenges. Here we asked whether Omnipose is able to detect cellular intoxication by Tre1. To this end, we incubated *S.* *proteamaculans* wild-type or a control strain expressing inactive Tre1 (*tre1*^*E415Q*^*)* with target *E.* *coli* cells and imaged these mixtures after 20 h. Owing to the improved segmentation accuracy of the Omnipose bact_phase model, we were able to include dense fields of view and incorporate ~300,000 cells in our analysis.

**Fig. 6:**
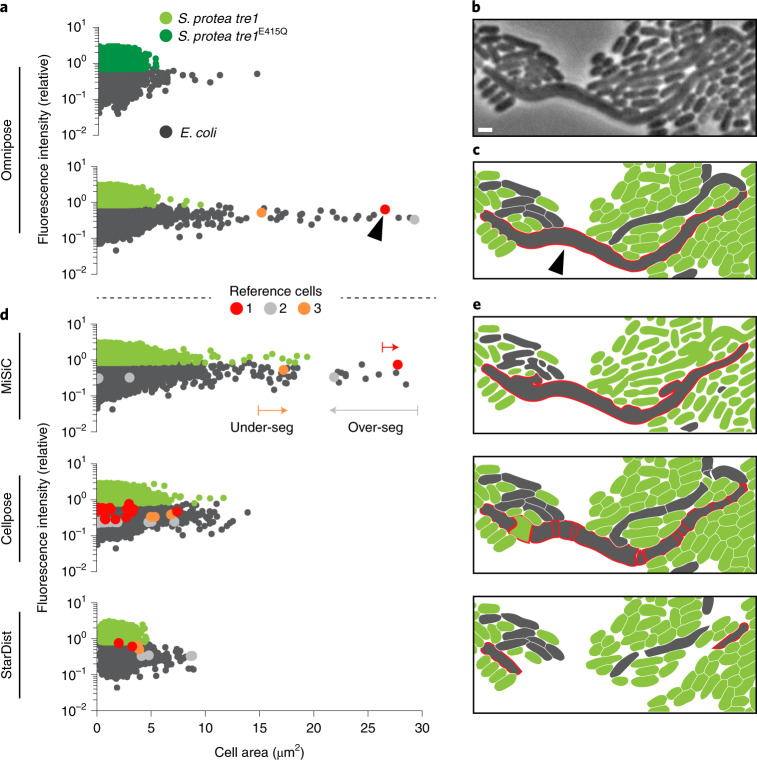
Omnipose facilitates the accurate identification of intoxicated *E.* *coli* cells. **a**, Fluorescence/area population profile according to Omnipose segmentation (bact_phase_omni) in control and experimental conditions. *K*-means clustering on GFP fluorescence distinguishes *S.* *proteamaculans tre1/tre1*^*E415Q*^ (light/dark green markers) from *E.* *coli* (gray markers). *n* = 209,000 cells in experimental population (*S.* *proteamaculans tre1*) and *n* = 85,260 cells in control group (*S.* *proteamaculans tre1*^*E415Q*^*)*. **b**, Example of extreme filamentation of *E.* *coli* in response to active Tre1. **c**, Omnipose accurately segments all cells in the image. Largest cell indicated with black arrow. **d**, MiSiC predicts large cell masks over both species. Cellpose (bact_phase_cp) and StarDist fail to predict any cells above 15 μm^2^. **e**, Example segmentation results highlighting typical errors encountered with MiSiC (under-segmentation), Cellpose (over-segmentation) and StarDist (incomplete masks). Mask mergers cause some *E.* *coli* to be misclassified as *S.* *proteamaculans*. Scale bar, 1 μm.

**Extended Data Fig. 10:**
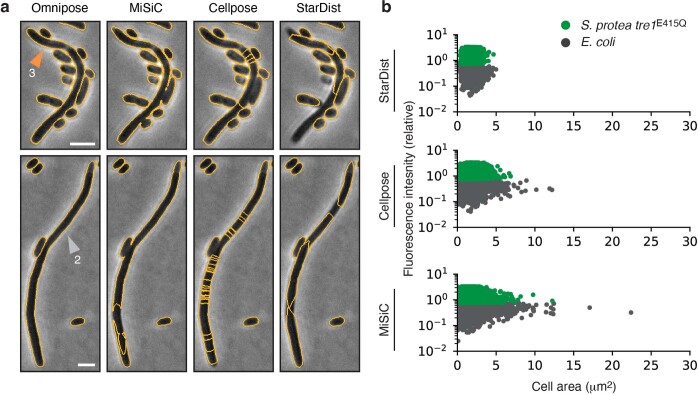
Example segmentation errors by StarDist, Cellpose and MiSiC of *E. coli* cells undergoing intoxication by *S. proteamaculans* Tre1. **(a)** Examples of segmentation failures demonstrated by each method. Cells 2 and 3 indicated with orange and gray arrows are the reference cells highlighted in Fig. 6a, d. Scale bars are 1 μm. (**b**) Control populations segmented by StarDist, Cellpose and MiSiC. Notably, Cellpose and MiSiC exhibit an enrichment of larger cells even in the control, a consequence of both under-segmented (merged) cells as well as fragments of over-segmented large cells.

Among the cells identified by Omnipose, we found that a small proportion were elongated and much larger than typical bacteria (Fig. 6a,b and Extended Data Fig. 10a). These cells were only detected in mixtures containing active Tre1 and the apparent failure of the cells to septate is consistent with the known FtsZ-inhibitory activity of the toxin. The *S.* *proteamaculans* strain background we employed in this work expresses the green fluorescent protein. Corresponding fluorescence images allowed us to unambiguously assign the enlarged cell population to *E.* *coli* (Fig. 6c). Next, we subjected the same images to cell segmentation with StarDist, Cellpose and MiSiC, the three top-performing algorithms in our initial survey. Each of these algorithms fail to identify this population of cells to high precision (Fig. 6d,e). Close inspection reveals three distinct modes of failure (Fig. 6e and Extended Data Fig. 10a,b). In the case of StarDist, elongated (non-star-convex) cells are split into multiple star-convex subsets that do not span the entire cell. Cellpose detects entire elongated cells but fragments them into a multitude of smaller masks. Conversely, MiSiC detects all cells but fails to properly separate them, thereby exaggerating the area measurement in many cases, including *E*. *coli* cells in the control experiment. These data illustrate how the enhanced cell segmentation performance of Omnipose can facilitate unique insights into microbiological systems.

## Discussion

We have designed Omnipose for use by typical research laboratories and we have made its source code, training data, and models publicly available (Supplementary Table 2). Thorough documentation demonstrating how to install and use Omnipose is also available (visit https://omnipose.readthedocs.io and see ‘Code availability’ section). For images similar to those represented in these models, we expect that researchers will not need to train models with new ground-truth data; however, users wishing to segment images not represented in our models (for example, cell type, imaging modality) should curate custom training data to obtain accurate results with Omnipose. Instructions for training and evaluating Omnipose are also provided within our documentation.

Confronted with the importance of segmentation accuracy to the success of work within our own laboratory, we were motivated to characterize the performance of several existing cell segmentation algorithms. Recent developments in deep learning have greatly improved these algorithms; however, important challenges remain^1,30^. Although isolated cells without cell-to-cell contact can be segmented with high precision by any of the packages that we tested, segmentation becomes vastly more challenging when cells form microcolonies, adopt irregular morphologies, or when images are composed of cells with assorted shapes and sizes. Such difficulties are compounded in timelapse studies, where the significance of segmentation errors often grows exponentially with time. Experimental design can help mitigate certain segmentation challenges; however, the recent emphasis on non-model organisms and microbial communities renders this an increasingly undesirable solution^42^.

This work provides a comprehensive side-by-side quantitative comparison of cell segmentation algorithm performance. As expected, machine-learning-based approaches outperform traditional image processing, yet insights into general image segmentation strategies can be gained from each of the methods we examined. Two of the six algorithms we tested utilize traditional image thresholding and watershed segmentation: Morphometrics and SuperSegger^13,23^. Each program tends to under-segment adjacent cells and over-segment large cells, behaviors previously linked to thresholding and watershed processes, respectively^1,43^. Given that SuperSegger was developed at least in part to mitigate these issues, we postulate that traditional image-segmentation approaches are ultimately limited to specialized imaging scenarios. Although we classify MiSiC as a DNN-based approach, this algorithm also relies on thresholding and watershed segmentation to generate cell masks from its network output^15^. The network output of MiSiC is more uniform than unfiltered phase-contrast images, yet this pre-processing does not fully abrogate the typical errors of thresholding and watershed segmentation.

A successful DNN-based algorithm is composed of a robust, consistent neural network output and an appropriate mask reconstruction process designed for this output. In the case of Mask R-CNN, bounding boxes for each cell are predicted along with a probability field that localizes a cell within its bounding box^44^. Masks are generated by iterating over each box and thresholding the probability field. Despite the widespread adoption of Mask R-CNN, we found this algorithm did not perform well in our study. Our results suggest that this is due to dense cell fields with overlapping bounding boxes, a feature known to corrupt the training process and produce poor network outputs for Mask R-CNN^45^. By contrast, the StarDist network makes robust predictions of its distance field, but it fails to assemble accurate cell masks because the cells in our dataset are not well approximated by star-convex polygons^26^. Likewise, the cell body and cell boundary outputs of MiSiC seem robust, but its watershed-based mask reconstruction is sensitive to defects in these outputs and can yield unreliable cell masks. The errors we encountered with Cellpose can be attributed to both neural network output and mask reconstruction. In Omnipose, we specifically addressed these two issues via the distance field and suppressed Euler integration, respectively, yielding a remarkably precise and generalizable image segmentation tool. Omnipose effectively leverages the strongest features of several of the DNN approaches we tested, namely the distance field of StarDist, the boundary field of MiSiC, and the mask reconstruction framework of Cellpose.

In this study, we emphasized morphological diversity, but we further accounted for differences in optical features between bacterial strains, slide preparation techniques, and microscope configurations. For example, bacterial strains exhibit a wide range of intrinsic contrast and internal structure, often exacerbated by antibiotic treatment or dense cell packing. Internal structure can cause over-segmentation, so we included many cells with this characteristic in our dataset. Additionally, the images in our ground-truth dataset originate from four different researchers using distinct microscopes, objectives, sensors, illumination sources, and acquisition settings. We further introduced extensive image augmentations that simulate variations in image intensity, noise, gamma, clipping, magnification, and bit depth (Methods).

Although Omnipose is designed to be trainable for any cell morphology or imaging modality, like all segmentation algorithms operating in 2D, Omnipose is limited in its ability to handle object overlap and self-contact (boundary intersection). Additionally, there are theoretical and practical limitations to the size of cells that it can accurately segment. Cells must be at least three pixels wide for the flow field to be well-defined at boundary pixels. A reasonable lower bound for cell size is therefore a 9-px square in 2D and a 27-voxel cube in 3D. Furthermore, we reason that the finite kernel size in our convolutional layers and number of downsampling steps in our U-net must present an upper limit to cell size that we have yet to quantify. In practice, we found that images with a mean cell diameter of 60 px or smaller are handled well during training by Omnipose. For images with cells larger than this, users may specify an average diameter to which the cells in all images are automatically rescaled before training. This solution may not be suitable for some applications and therefore more work is needed to modify our U-net implementation to achieve native segmentation at arbitrarily high resolutions. When segmenting new images, users are able to manually define a rescaling factor to bring cells into an acceptable size range or estimate this rescaling factor using a SizeModel, a feature inherited from Cellpose and improved by our mean diameter metric (Methods). Omnipose in 3D avoids issues of cell overlap, but the problem of self-intersecting boundaries remains. It is conceivable that the use of a flow field could be leveraged to define such boundaries, making Omnipose a promising candidate for overcoming this widespread limitation of segmentation algorithms. A dearth of 3D ground-truth data hampers the training and evaluation of all DNN-based algorithms. Manual annotation of 3D volumes is considerably more difficult than 2D images, leading to errors that corrupt training.

We anticipate that the high performance of Omnipose across varied cellular morphologies and modalities may unlock information from microscopy images that was previously inaccessible. All bacteria appear dark under phase contrast, so images deriving from natural microbial communities could be segmented by phase contrast and accurately characterized with regard to internal structure, autofluorescence, and morphology at the single-cell level. These data could be used to estimate diversity, a methodology that would complement existing sequencing-based metrics^46^. It is worth noting that phenotypic diversity often exceeds genetic diversity^47^; therefore, even in a relatively homogeneous collection of organisms, precise segmentation could allow classes representing distinct states to be identified. A microscopy-based approach also offers the opportunity to characterize spatial relationships between cells, information that is exceptionally difficult to recover in most biomolecular assays.

## Methods

### Phase-contrast and fluorescence microscopy

In-house imaging was performed on a Nikon Eclipse Ti-E wide-field epi-fluorescence microscope, equipped with a sCMOS camera (Hamamatsu) and X-cite LED for fluorescence imaging. We imaged through ×60 and ×100 1.4 NA oil-immersion PH3 objectives. The microscope was controlled by NIS-Elements v.3.30.02. Cell samples were spotted on a 3% (w/v) agarose pad placed on a microscope slide. The microscope chamber was heated to 30 °C or 37 °C when needed for timelapse experiments.

Several images in our dataset were taken by two other laboratories using three distinct microscope/camera configurations. The Brun laboratory provided images of *C.* *crescentus* acquired on a Nikon Ti-E microscope equipped with a Photometrics Prime 95B sCMOS camera. Images were captured through a ×60 Plan Apo λ ×100 1.45 NA oil Ph3 DM objective. The Wiggins laboratory provided *E.* *coli* and *A.* *baylyi* timelapses from both a Nikon Ti-E microscope using NIS-Elements v.4.10.01 as well as a custom-built tabletop microscope using Micro-Manager v.1.4, both of which are described in previous studies^48,49^.

### *C.elegans* data preparation

We obtained a 1,000-frame timelapse of *C.* *elegans* from the Wormpose^38^ GitHub (https://github.com/iteal/wormpose_data) adapted from the Open Worm Movement database^39^, which is inaccessible at the time of writing. We also utilized BBBC010 (ref. ^40^) (https://bbbc.broadinstitute.org/c-elegans-livedead-assay-0), a set of 100 images containing live and dead *C.* *elegans*. These images were manually cropped to select regions of each image without *C.* *elegans* overlaps. For both of these datasets, images were initially segmented with Omnipose to select foreground, automatically cropped to select individual *C.* *elegans* or clusters of *C. elegans* and then packed into ensemble images for efficient annotation, training and testing following the same procedures described below for our bacterial datasets.

For high-resolution *C.* *elegans* images, one gravid non-starved nematode growth medium plate of wild-type *C.* *elegans* (QZ0) was resuspended in 1 ml M9 defined buffer. The worm suspension was pelted by quick spinning and resuspended in 100 μl fresh M9 buffer. Then, 20 μl of the pellet was placed on agar pads (0.3% agar, SeaKem) and mounted on regular microscope slides (25 mm × 75 mm). The 20-μl drops were left to dry by approximately 50% of their volume at room temperature, allowing worms to arrange longitudinally, before a glass coverslip (22 mm × 22 mm) was placed on top. The sample was imaged using a Andor Dragonfly Spinning disk confocal mounted on a Nikon TiE2 microscope at ×15 magnification (×10/0.25 NA Nikon objective and ×1.5 camera magnification) in the brightfield channel. For each field of view, a Z stack of seven frames centered on the worm focal plane and spanning over approximately 85 μm (12.14-μm spacing between frames) was acquired.

### *A.thaliana* data preparation

This specific subset of the PlantSeg dataset^22^ was chosen because it represented diverse morphologies and because other subsets of their published ground truth were not in accessible formats. Three folders were provided: test, train and val. We first combined the test and val volumes into a single test dataset. The PlantSeg algorithm seems to exclude regions labeled by 0 as during training, whereas 1 denotes background. This is incompatible with Omnipose and most other algorithm training pipelines. We therefore discarded images with the label 0 from our training set and subtracted 1 so that the remaining images conformed to the convention of 0 for background. The final training set consists of Video 1 timepoints 3, 9, 35, 40, 45 and 49. The published test dataset consists of Video 2 frames 10 and 20 and Video 1 frames 4, 6, 30 and 45. Frame 45 is mistakenly duplicated from the training set, so we discarded it from our test metrics. Only frame 30 of Video 1 contained no exclusion zones (label 0), so we present both the performance on this subset alone as well as the performance on the full test dataset (Video 1 frames 4, 6 and 30 and Video 2 frames 10 and 20) (Fig. 6e). For the purposes of computing performance, exclusion zones are treated as background and therefore all predicted labels in these regions significantly decrease the JI for all algorithms at all IoU thresholds regardless of segmentation accuracy elsewhere. The images and ground truth were downsampled by a factor of three to allow full cell cross-sections to be loaded onto the GPU during training (see below section, Omnipose in higher dimensions), with linear interpolation on images and nearest-neighbor interpolation on the ground-truth masks.

### Bacterial sample preparation

To image antibiotic-induced phenotypes, cells were grown without antibiotics overnight in LB, back-diluted and spotted on agarose pads with 50 µg ml^−1^ A22 or 10 µg ml^−1^ cephalexin. Timelapses were captured of *E.* *coli* DH5α and *S.* *flexneri* M90T growing on these pads. *E.* *coli* CS703-1 was back-diluted into LB containing 1 µg ml^−1^ aztreonam and spotted onto a pad without antibiotics^50^. Cells constitutively expressed GFP to visualize cell boundaries.

*H.* *pylori* LSH100 grown with and without aztreonam was provided by the Salama laboratory^51,52^. Samples were fixed and stained with Alexa Fluor 488 to visualize the cell membrane. Images were taken on LB pads. The typical technique of allowing the spot to dry on the pad caused cells to curl up on themselves, therefore our images were taken by placing the coverslip on the pad immediately after spotting and applying pressure to force out excess medium.

*C.* *crescentus* was cultivated and imaged by the Brun laboratory^53,54^. Cells were grown in PYE, washed twice in water before 1:20 dilution in HIGG medium and grown at 26 °C for 72 h. Cells were spotted on a 1% agarose PYE pads before imaging.

*S.* *pristinaespiralis* NRRL 2958 was grown using the following medium recipe: yeast extract 4 g l^−1^, malt extract 10 g l^−1^, dextrose 4 g l^−1^ and agar 20 g l^−1^. This medium was used to first culture the bacteria in liquid overnight and then on a pad under the microscope. This strain forms aggregates in liquid medium, so these aggregates were allowed to grow for several hours on a slide in a heated microscope chamber until we could see individual filaments extending from the aggregates. Fields of view were selected and cropped to exclude cell overlaps. Autofluorescence was captured to aid in manual segmentation.

Mixtures of *S.* *proteamaculans* attTn7::Km-gfp *tre1* or *tre1*^*E415Q*^ and *E.* *coli* were spotted on a PBS pad to prevent further growth. Phase-contrast images of the cells were acquired before and after a 20-h competition on a high-salt LB plate. Fluorescence images in the GFP channel were also acquired to distinguish *S.* *proteamaculans* from unlabeled *E.* *coli*.

All other individual strains in Supplementary Table 1 were grown overnight, diluted 1:100 into fresh LB medium and grown for 1–3 h before imaging. Mixtures were made by combining back-diluted cells roughly 1:1 by OD_600_.

### Manual image annotation

Manual annotation began with loading images into MATLAB, normalizing the channels, registering the fluorescence channel(s) to brightfield (when applicable) and producing boundary-enhanced versions of brightfield and fluorescence. Where possible, fluorescence data were primarily used to define cell boundaries (not available in the *C.* *elegans* dataset acquired online). In addition to a blank channel to store manual labels, all processed phase and fluorescence images were then automatically loaded as layers into an Adobe Photoshop document. We used four to six unique colors and the Pencil tool (for pixel-level accuracy and no blending) to manually define object masks. Due to the four-color theorem^55^, this limited palette was sufficient to clearly distinguish individual object instances from each other during annotation. This color simplification enabled faster manual annotation by reducing the need to select new colors. It also eliminated the confusion caused by the use of similar but distinct colors in adjacent regions, which we suspect is the cause for the misplaced mask pixels that we observed in other datasets (for example, cyto2). Consistency was ensured by enlisting a single individual to perform all manual annotations.

The cell label layer was then exported as a PNG from Photoshop, read back into MATLAB and converted from the repeating *n*-color labels to a standard 16-bit integer label matrix, where each object is assigned a unique integer from 1 to the number of cells (background is 0). Because integer labels cannot be interpolated, we then performed a non-rigid image registration of the brightfield channel to the binary label mask to achieve better brightfield correlation to ground-truth masks. All images in our ground-truth dataset have been registered in this manner.

### Choosing segmentation algorithms

Three main factors contributed to the choice of algorithms highlighted in this study: (1) specificity to bacterial phase-contrast images; (2) success and community adoption, especially for bioimage segmentation; and (3) feasibility of installation, training and use. It is noteworthy that criterion (1) only influenced the choice of non-DNN algorithms because they are generally modality-, scale- and subject-specific in their design. DNN approaches can generally be trained on arbitrary sets of images. With the exception of MiSiC, none of the DNN-based approaches we chose were specifically designed for (or substantively trained on) bacterial phase-contrast images.

SuperSegger, Morphometrics and MiSiC were selected because they specifically targeted the problem of bacterial phase-contrast segmentation^13,15,23^. Other bacteria-focused packages do exist, such as BactMAP, BacStalk, Cellprofiler, CellShape, ColiCoords, Cytokit, MicroAnalyzer, MicrobeJ, Oufti and Schnitzcells; however, these incorporate limited new segmentation solutions and instead aim to provide tools for single-cell analysis such as lineage tracing and protein tracking^8,9,14,18–20,25,56–58^. Furthermore, the segmentation that these programs perform depends broadly on thresholding and watershed techniques; therefore, Morphometrics is a reasonable proxy for their segmentation capabilities. We were unable to locate code or training data for BASCA at the time of writing^11^. Ilastik is a popular interactive machine-learning tool for bioimage segmentation, but training it using a manual interface was not feasible on a large and diverse dataset such as our own^21^.

Among DNN approaches, Mask R-CNN was selected because it is a popular architecture for handling typical image segmentation tasks. It was also used in the segmentation and tracking package Usiigaci^24^. U-Net architectures have been implemented in a number of algorithms, including DeLTA, PlantSeg, MiSiC, StarDist and Cellpose^12,15,17,22,26^. DeLTA was not included in this study because it operates similarly to MiSiC and was designed specifically for mother machine microfluidics analysis. DeLTA 2.0 was recently released to additionally segment confluent cell growth on agarose pads, but it remains quite similar to MiSiC in implementation^59^. PlantSeg could, in principle, be trained on bacterial micrographs, but we determined that its edge-focused design meant to segment bright plant cell wall features would not offer any advancements over the remaining U-net methods that we tested.

### Training and tuning segmentation algorithms

All segmentation algorithms have tunable parameters to optimize performance on a given dataset. These include pre-processing such as image rescaling (often to put cells into a particular pixel diameter range), contrast adjustment, smoothing and noise addition. Morphometrics and SuperSegger were manually tuned to give the best results on our benchmarking dataset. The neural network component of SuperSegger was not retrained on our data, as this is a heavily manual process involving toggling watershed lines on numerous segmentation examples. DNN-based algorithms are automatically trained using our dataset and the scripts we used to do so are available in our GitHub repository. We adapted our data for MiSiC by transforming our instance labels into interior and boundary masks. Training documentation for MiSiC is not published. Training and evaluation parameters for MiSiC were tuned according to correspondence with the MiSiC authors. Cellpose and StarDist were trained with the default parameters provided in their documentation. StarDist has an additional tool to optimize image pre-processing parameters on our dataset, which we utilized.

### Evaluating segmentation algorithms

All algorithms were evaluated on our benchmarking dataset with manually or automatically optimized parameters. We provide both the raw segmentation results for all test images by each tested algorithm as well as the models and model-training scripts required to reproduce our results. Before evaluating IoU or JI, small masks at image boundaries were removed for both the ground-truth and predicted masks. IoU and JI are calculated on a per-image basis and, where shown, are averaged with equal weighting over the image set or field of view.

Our new metric, the number of segmentation errors per cell, was calculated by first measuring the fraction of each predicted cell that overlaps with each ground-truth cell. A predicted cell is assigned to a ground-truth cell if the overlap ratio is ≥0.75, meaning that at least three quarters of the predicted cell lies within the ground-truth cell. If several predicted cells are matched to a ground-truth cell, the number of surplus matches is taken to be the number of segmentation errors. If no cells are matched to a ground-truth cell, then the error is taken to be 1.

### Statistics and reproducibility

The neural network models in this study are trained using seeded pseudo-random shuffling of labeled data into batches. This means that all models converge to precisely the same weights and biases given the same dataset and hyperparameters. Neural network evaluation is also entirely deterministic. For these reasons, no segmentation results in this study are associated with repetition or statistics.

All segmentation results and figures in this study can be programmatically reproduced using the figure scripts in our GitHub repository (see ‘Code availability’ section). Figure micrographs are exported programmatically by manually selected labels within our scripts and automatic cropping around selected labels. Although some pre-filtering by rough statistical metrics may have been used to help select example cells, specific examples were ultimately selected for illustrative purposes and are intended only to be qualitatively representative of aspects such as cell phenotype or algorithm failure modes.

### Leveraging Omnipose to accelerate manual annotation

Omnipose was periodically trained on our growing dataset to make initial cell labels. These were converted into an *n*-color representation and loaded into Photoshop for manual correction. A subset of our cytosol GFP channels were sufficient for training Omnipose to segment based on fluorescence and the resulting trained model enabled higher-quality initial cell labels for GFP-expressing samples than could be achieved from intermediate phase-contrast models (for example, *V.* *cholerae*).

### Defining the Omnipose prediction classes

Omnipose predicts four classes: two flow components, the distance field and a boundary field. Our distance field is found by solving the eikonal equation

$$\left| {\vec \nabla \phi \left( {\vec x} \right)} \right| = \frac{1}{{f\left( {\vec x} \right)}}$$

where *f* represents the speed at a point $\vec x$. The Godunov upwind discretization of the eikonal equation is

$$\begin{array}{l}\left( {\frac{{\max \left( {\phi _{i,j} - \min \left( {\phi _{i - 1,j},\phi _{i + 1,j}} \right),0} \right)}}{{{{\Delta }}x}}} \right)^2 + \left( {\frac{{\max \left( {\phi _{i,j} - \min \left( {\phi _{1,j - 1},\phi _{i,j + 1}} \right),0} \right)}}{{{{\Delta }}y}}} \right)^2 = \frac{1}{{f_{i,j}}}\end{array}$$

Our solution to this equation is based on the Improved FIM Algorithm 1.1 (ref. ^34^), as follows. Our key contribution to this algorithm is the addition of ordinal sampling to boost both convergence and smoothness of the final distance field.

#### 2D update function for *ϕ*_*i,j*_ on a Cartesian grid

1. Find neighboring points for cardinal axes (Δ*x* = Δ*y* = *δ*):
  $$\phi ^{{{{\mathrm{minx}}}}} = \min \left( {\phi _{i - 1,j},\phi _{i + 1,j}} \right),\quad \phi ^{{{{\mathrm{miny}}}}} = \min \left( {\phi _{i,j - 1},\phi _{i,j + 1}} \right)$$
2. Find neighboring points for ordinal axes ($\hat x \cdot \hat a = \hat y \cdot \hat b = \frac{{\sqrt 2 }}{2},\frac{{{{\Delta }}a}}{{{{\Delta }}x}} = \frac{{{{\Delta }}b}}{{{{\Delta }}y}} = \sqrt 2 \delta$):
  $$\phi ^{{{{\mathrm{mina}}}}} = \min \left( {\phi _{i - 1,j - 1},\phi _{i + 1,j + 1}} \right),\quad \phi ^{{{{\mathrm{minb}}}}} = \min \left( {\phi _{i + 1,j - 1},\phi _{i - 1,j + 1}} \right)$$
3. Calculate update along cardinal axes:
  $${{\mathbf{if}}}\,\left| {\phi ^{{{{\mathrm{minx}}}}} - \phi ^{{{{\mathrm{miny}}}}}} \right| > \frac{{\sqrt 2 \delta }}{{f_{i,j}}}:$$
  $$U^{xy} = \min \left( {\phi ^{{{{\mathrm{minx}}}}},\phi ^{{{{\mathrm{miny}}}}}} \right) + \frac{\delta }{{f_{i,j}}}$$
  **else**:
  $$U^{xy} = \frac{1}{2}\left( {\phi ^{{{{\mathrm{minx}}}}} + \phi ^{{{{\mathrm{miny}}}}} + \sqrt {2\left( {\frac{\delta }{{f_{i,j}}}} \right)^2 - \left( {\phi ^{{{{\mathrm{minx}}}}} - \phi ^{{{{\mathrm{miny}}}}}} \right)^2} } \right)$$
4. Calculate updat e along ordinal axes:
  $${{\mathbf{if}}}\,\left| {\phi ^{{{{\mathrm{mina}}}}} - \phi ^{{{{\mathrm{minb}}}}}} \right| > \frac{{2\delta }}{{f_{i,j}}}:$$
  $$U^{ab} = \min \left( {\phi ^{{{{\mathrm{mina}}}}},\phi ^{{{{\mathrm{minb}}}}}} \right) + \frac{{\sqrt 2 \delta }}{{f_{i,j}}}$$
  **else**:
  $$U^{ab} = \frac{1}{2}\left( {\phi ^{{{{\mathrm{mina}}}}} + \phi ^{{{{\mathrm{minb}}}}} + \sqrt {4\left( {\frac{\delta }{{f_{i,j}}}} \right)^2 - \left( {\phi ^{{{{\mathrm{mina}}}}} - \phi ^{{{{\mathrm{minb}}}}}} \right)^2} } \right)$$
5. Update with geometric mean:

$$\phi _{i,j} = \sqrt {U^{xy}U^{ab}}$$

This update rule is repeated until convergence (Extended Data Fig. 5). We take *δ* = *f*_*i,j*_ to obtain the signed distance field used in Omnipose. The flow field components are defined by the normalized gradient of this distance field *ϕ*. The boundary field is defined by points satisfying 0 < *ϕ* < 1. For network prediction, the boundary map is converted to the logits (inverse sigmoid) representation, such that points in the range [0,1] are mapped to [−5,5]. For consistent value ranges across prediction classes, the flow components are multiplied by 5 and all background values of the distance field (*ϕ*=0) are set to −5.

### Omnipose network architecture

The DNN used for Omnipose is a minor modification of that used in Cellpose: a U-net architecture with two residual blocks per scale, each with two convolutional layers^12^. Omnipose introduces a dropout layer before the densely connected layer^60^, which we incorporated into the shared Cellpose and Omnipose architecture moving forward (see resnet_torch.py); however, Cellpose models utilized in this study are trained without dropout.

### Omnipose in higher dimensions

The network architecture described above is implemented in PyTorch and is generalized to 3D by taking a ‘dimension’ argument that chooses, for example, Conv3D instead of Conv2D. This is a key component of Omnipose that is not fully generalized to arbitrary dimension (ND) segmentation because it depends on these explicit 2D and 3D PyTorch classes. Custom implementations of these classes (for example, ConvND) will be needed for 4D segmentation, which we envision being highly useful for processing 3D timelapses. The Omnipose methods for generating boundary, distance, and flow are completely dimension-agnostic, as is the mask reconstruction algorithm; however, a dependency, the edt package, needs to be generalized to ND. It is used both to compute boundaries (exact distance of 1) and to estimate the number of iterations required for our smooth distance function to converge (linear scaling of the maximum of the exact distance). Workarounds for each of these uses can be found if edt is not updated. Boundaries can be found by appropriately generalized binary hit–miss operators or ND mask-erosion difference maps. The smooth distance could simply be set to terminate after it has reached convergence.

The large memory footprint of 3D volumes also motivated us to use DataParallel (models.py) to allow for multi-GPU training. We note that DistributedDataParallel is a more efficient (but far less convenient) implementation that might be needed to train on batches of larger volumes. The default 3D volumes after argumentations are currently 84 × 84 × 84 and take up about 12 GB of VRAM each. The size of volumes should be at least the diameter of an average cell (ideally much larger), so this can be tuned according to the dataset. We implemented a –tyx flag for specifying the size of volumes crops to be used in training (for example, ‘50,50,50’).

### Rescaling flow field by divergence

During training, the ground-truth data are augmented by a random affine transformation. The original implementation, and the one which yields the best results, linearly interpolates the transformed field. This reduces the magnitude of the otherwise normalized field in regions of divergence (at boundaries and skeletons). A renormalized field (obtained either from the transformed field or as the normalized gradient of the transformed heat distribution) often has artifacts at cell boundaries and skeletons, so the interpolated field effectively reduces the influence of these artifacts on training. We reason that this feature explains the superior performance of interpolated field training over renormalized fields, despite the latter being the nominal goal of the algorithm.

Pixels at cell boundaries, however, consequently do not move far (<1 px) under Euler integration due to the low magnitude of the predicted field at cell boundaries. Our solution in Omnipose is to rescale the flow field by the magnitude of the divergence. The divergence is most positive at the cell boundaries (where pixels need to move) and most negative at cell skeletons (where pixels need to stop). We therefore rescale the divergence from 0 to 1 and multiply the normalized flow field by this new magnitude map. This forces boundary pixels of neighboring cells to quickly diverge and allow for accurate pixel clustering to obtain the final segmentation.

### New diameter metric

The size models of Cellpose are trained to estimate the average cell ‘diameter’, taken to be the diameter of the circle of equivalent area:

1 $$d=2{\mathrm{R}}=2 \sqrt{\frac{A}{\pi}}$$

This metric as a basis for rescaling is problematic when cells are growing in length but not width (Extended Data Fig. 1). Log-phase bacterial cell area grows exponentially with time and so too does the scale factor, eventually resulting in a rescaled image that is too small for Cellpose to segment.

The average of the distance field, however, does not change for filamentous bacteria, as the width and therefore the distance to the closest boundary, remains constant. To define a formula consistent with the previous definition in the case of a circular cell, we consider mean of the distance field over the cell:

$$\bar \phi = \frac{1}{{\pi R^2}}{\int}_0^{2\pi } {{\int}_0^R {\left( {R - r} \right)rdrd\theta = \frac{1}{{\pi R^2}}\left( {\frac{\pi }{3}R^3} \right) = \frac{R}{3}} }$$

This allows us to define a new ‘effective diameter’ as

2 $$d = 2R = 6\bar \phi$$

Aside from agreeing with the previous scaling method (*) for round morphologies, this definition exhibits excellent consistency across time (Extended Data Fig. 1). This consistency is also critical for training on datasets with wide distributions in cell areas that require rescaling, such as the Cellpose datasets. A SizeModel can be trained using the Omnipose metric for automatic size estimation and image rescaling. Finally, the raw distance field output of Omnipose can directly be used directly in (**) to estimate average cell diameter, which is used in our code to automatically toggle on features that improve mask reconstruction performance for small cells.

### Gamma augmentation

To make the network robust against changes in exposure/contrast, the training images are now raised to a random power (γ) between 0.5 and 1.25, simulating the varying levels of contrast that are observed experimentally with different light sources, objectives, and exposure times.

### Alleviating class imbalance

Class imbalance remains a challenge in many machine-learning applications^61^. In our dataset, foreground pixels (cells) take up anywhere from 1% to 75% of a given training image, with the rest being background pixels that the network must only learn to ignore (assign a constant output of −5 for distance and boundary logits). We implemented several changes to the loss function to emphasize foreground objects, including weighting by the distance field and averaging some loss terms only over foreground pixels. Our training augmentation function also attempts many random crop and resizing passes until a field of view with foreground pixels is selected (this may take several attempts for sparse images but adds very little time to training).

### Image normalization

To manage different image exposure levels, Cellpose automatically rescales images such that pixels in the first percentile of intensity are set to 0 and those in the 99th percentile are sent to 1. This percentile rescaling is preferred over blind min–max rescaling because bubbles or glass can cause small bright spots in the image; however, we found that images containing single cells (low intensity) in a wide field of media (high intensity) would become badly clipped due to the foreground-background class imbalance. To solve this, we changed the percentile range to be from 0.01 to 99.99.

### Reporting summary

Further information on research design is available in the Nature Research Reporting Summary linked to this article.

## Online content

Any methods, additional references, Nature Research reporting summaries, source data, extended data, supplementary information, acknowledgements, peer review information; details of author contributions and competing interests; and statements of data and code availability are available at 10.1038/s41592-022-01639-4.

## Supplementary information

Supplementary Information Supplementary Table references.

Reporting Summary

Supplementary Table 1 Bacterial strains and ground truth annotation.

Supplementary Table 2 Trained Omnipose and Cellpose models.

## Data Availability

Bacterial phase-contrast and fluorescence image sets generated in this study, unpublished bacterial phase-contrast images provided by Y. Brun, and unpublished minimum projection brightfield images of *C.* *elegans* provided by L. Rappez are available in our OSF repository https://osf.io/xmury/ under the CC BY-NC 3.0 license. Additional *C.* *elegans* images were sourced from a selection of the Open Worm Movement dataset by the Wormpose project at https://github.com/iteal/wormpose_data and from the Broad Bioimage Benchmark Collection set BBBC010 at https://bbbc.broadinstitute.org/c-elegans-livedead-assay-0, both of which are available under the CC-4.0 license. Files deriving from our processing and labeling of these data are available in our OSF repository https://osf.io/xmury/ under the CC BY-NC 3.0 license. *A.* *thaliana* image volumes were sourced from the PlantSeg OSF database at https://osf.io/uzq3w/. The cyto2 dataset was sourced from http://www.cellpose.org/dataset.

## Code availability

For installation instructions and source code, see our documentation at https://omnipose.readthedocs.io and our GitHub repository at https://github.com/kevinjohncutler/omnipose. Standalone apps for Windows, macOS and Linux are available in our OSF repository https://osf.io/xmury/. Data processing and analysis scripts for this study were written in MATLAB 2021a and Python ≥3.8.5. All scripts are available at https://github.com/kevinjohncutler/omnipose/tree/main/figures. Figure panels were assembled in Adobe Illustrator v.26.2.1. The algorithms used in comparison to Omnipose are sourced as follows: SuperSegger v.1.1, https://github.com/wiggins-lab/SuperSegger; Cellpose v.1.0.2, https://github.com/MouseLand/cellpose; StarDist v.0.7.2, https://github.com/stardist/stardist; MiSiC v.1.0.3, https://github.com/pswapnesh/misic; Mask R-CNN from Torchvision v.0.12.0, https://github.com/pytorch/vision; and Morphometrics v.1.102, https://simtk.org/projects/morphometrics.

## References

[1] Jeckel, H. & Drescher, K. Advances and opportunities in image analysis of bacterial cells and communities. *FEMS Microbiol Rev*10.1093/femsre/fuaa062 (2021). 10.1093/femsre/fuaa062 PMC8371272 33242074

[2] Bali, A. & Singh, S. N. A review on the strategies and techniques of image segmentation. In *2015 Fifth International Conference on Advanced Computing & Communication Technologies* 113–120 (2015).

[3] Lucas AM (2021). Open-source deep-learning software for bioimage segmentation. Mol. Biol. Cell.

[4] Kysela DT, Randich AM, Caccamo PD, Brun YV (2016). Diversity takes shape: understanding the mechanistic and adaptive basis of bacterial morphology. PLoS Biol..

[5] Jones SE, Elliot MA (2018). ‘Exploring’ the regulation of *Streptomyces* growth and development. Curr. Opin. Microbiol..

[6] Caccamo PD, Brun YV (2018). The molecular basis of noncanonical bacterial morphology. Trends Microbiol..

[7] Behera B (2019). Emerging technologies for antibiotic susceptibility testing. Biosens. Bioelectron..

[8] Paintdakhi A (2016). Oufti: an integrated software package for high-accuracy, high-throughput quantitative microscopy analysis. Mol. Microbiol..

[9] Ducret A, Quardokus EM, Brun YV (2016). MicrobeJ, a tool for high throughput bacterial cell detection and quantitative analysis. Nat. Microbiol.

[10] Tropini C, Earle KA, Huang KC, Sonnenburg JL (2017). The gut microbiome: connecting spatial organization to function. Cell Host Microbe.

[11] Balomenos AD (2017). Image analysis driven single-cell analytics for systems microbiology. BMC Syst. Biol..

[12] Stringer C, Wang T, Michaelos M, Pachitariu M (2021). Cellpose: a generalist algorithm for cellular segmentation. Nat. Methods.

[13] Stylianidou S, Brennan C, Nissen SB, Kuwada NJ, Wiggins PA (2016). SuperSegger: robust image segmentation, analysis and lineage tracking of bacterial cells. Mol. Microbiol..

[14] van Raaphorst R, Kjos M, Veening JW (2020). BactMAP: An R package for integrating, analyzing and visualizing bacterial microscopy data. Mol. Microbiol..

[15] Panigrahi, S. et al. Misic, a general deep learning-based method for the high-throughput cell segmentation of complex bacterial communities. *eLife*10.7554/eLife.65151 (2021). 10.7554/eLife.65151 PMC8478410 34498586

[16] Bannon D (2021). DeepCell Kiosk: scaling deep learning-enabled cellular image analysis with Kubernetes. Nat. Methods.

[17] Lugagne JB, Lin H, Dunlop MJ (2020). DeLTA: Automated cell segmentation, tracking, and lineage reconstruction using deep learning. PLoS Comput. Biol..

[18] Smit JH, Li Y, Warszawik EM, Herrmann A, Cordes T (2019). ColiCoords: a Python package for the analysis of bacterial fluorescence microscopy data. PLoS ONE.

[19] Czech E, Aksoy BA, Aksoy P, Hammerbacher J (2019). Cytokit: a single-cell analysis toolkit for high dimensional fluorescent microscopy imaging. BMC Bioinf..

[20] McQuin C (2018). CellProfiler 3.0: Next-generation image processing for biology. PLoS Biol..

[21] Berg S (2019). ilastik: interactive machine learning for (bio)image analysis. Nat. Methods.

[22] Wolny, A. et al. Accurate and versatile 3D segmentation of plant tissues at cellular resolution. *eLife*10.7554/eLife.57613 (2020). 10.7554/eLife.57613 PMC7447435 32723478

[23] Ursell T (2017). Rapid, precise quantification of bacterial cellular dimensions across a genomic-scale knockout library. BMC Biol..

[24] Tsai HF, Gajda J, Sloan TFW, Rares A, Shen A (2019). Usiigaci: Instance-aware cell tracking in stain-free phase contrast microscopy enabled by machine learning. SoftwareX.

[25] Reiner, J., Azran, G. & Hyams, G. MicroAnalyzer: a Python tool for automated bacterial analysis with fluorescence microscopy. Preprint at *arXiv* https://arxiv.org/abs/2009.12684 (2020).

[26] Schmidt, U. et al. in *Medical Image Computing and Computer Assisted Intervention* (Springer, 2018).

[27] He, K., Gkioxari, G., Dollar, P. & Girshick, R. Mask R-CNN. Preprint at *arXiv* https://arxiv.org/abs/1703.06870 (2018). 10.1109/TPAMI.2018.2844175 29994331

[28] Shal K, Choudhry MS (2021). Evolution of deep learning algorithms for MRI-based brain tumor image segmentation. Crit. Rev. Biomed. Eng..

[29] Bean GJ (2009). A22 disrupts the bacterial actin cytoskeleton by directly binding and inducing a low-affinity state in MreB. Biochemistry.

[30] Laine RF, Arganda-Carreras I, Henriques R, Jacquemet G (2021). Avoiding a replication crisis in deep-learning-based bioimage analysis. Nat. Methods.

[31] Taha AA, Hanbury A (2015). Metrics for evaluating 3D medical image segmentation: analysis, selection, and tool. BMC Med. Imaging.

[32] Lu W (2017). Unsupervised sequential outlier detection with deep architectures. IEEE Trans. Image Process..

[33] Sethian JA, Vladimirsky A (2001). Ordered upwind methods for static Hamilton-Jacobi equations. Proc. Natl Acad. Sci. USA.

[34] Huang, Y. Improved fast iterative algorithm for eikonal equation for GPU computing. Preprint at *arXiv* https://arXiv:2106.15869v3 (2021).

[35] Ester, M., Kreigel, H. P., Sander, J. & Xu, X. A density-based algorithm for discovering clusters in large spatial databases with noise. In *Proc. 2nd Int. Conf. Knowl. Discov. Data Min.* 226–231 (1996).

[36] Gitai Z (2009). New fluorescence microscopy methods for microbiology: sharper, faster, and quantitative. Curr. Opin. Microbiol..

[37] Girard LR (2007). WormBook: the online review of *Caenorhabditis elegans* biology. Nucleic Acids Res..

[38] Hebert L, Ahamed T, Costa AC, O’Shaughnessy L, Stephens GJ (2021). WormPose: image synthesis and convolutional networks for pose estimation in *C.* *elegans*. PLoS Comput. Biol..

[39] Javer A (2018). An open-source platform for analyzing and sharing worm-behavior data. Nat. Methods.

[40] Ljosa V, Sokolnicki KL, Carpenter AE (2012). Annotated high-throughput microscopy image sets for validation. Nat. Methods.

[41] Ting SY (2018). Bifunctional immunity proteins protect bacteria against FtsZ-targeting ADP-ribosylating toxins. Cell.

[42] Cusick, J. A., Wellman, C. L. & Demas, G. E. The call of the wild: using non-model systems to investigate microbiome-behaviour relationships. *J. Exp. Biol*. 10.1242/jeb.224485 (2021). 10.1242/jeb.224485 PMC8180253 33988717

[43] Wang Z (2019). Cell segmentation for image cytometry: advances, insufficiencies, and challenges. Cytom. A.

[44] He C (2021). Genome-resolved metagenomics reveals site-specific diversity of episymbiotic CPR bacteria and DPANN archaea in groundwater ecosystems. Nat. Microbiol.

[45] Looi, S. rotated_maskrcnn. *GitHub* https://github.com/mrlooi/rotated_maskrcnn (2019).

[46] Bharti R, Grimm DG (2021). Current challenges and best-practice protocols for microbiome analysis. Brief. Bioinform..

[47] Smits WK, Kuipers OP, Veening JW (2006). Phenotypic variation in bacteria: the role of feedback regulation. Nat. Rev. Microbiol..

[48] Bailey J (2019). Essential gene deletions producing gigantic bacteria. PLoS Genet..

[49] Cass JA, Stylianidou S, Kuwada NJ, Traxler B, Wiggins PA (2017). Probing bacterial cell biology using image cytometry. Mol. Microbiol..

[50] Meberg BM, Sailer FC, Nelson DE, Young KD (2001). Reconstruction of *Escherichia coli* mrcA (PBP 1a) mutants lacking multiple combinations of penicillin binding proteins. J. Bacteriol..

[51] Lowenthal AC (2009). Functional analysis of the *Helicobacter pylori* flagellar switch proteins. J. Bacteriol..

[52] Taylor, J. A. et al. Distinct cytoskeletal proteins define zones of enhanced cell wall synthesis in *H. pylori*. *eLife*10.7554/eLife.52482 (2020). 10.7554/eLife.52482 PMC7012605 31916938

[53] Evinger M, Agabian N (1977). Envelope-associated nucleoid from *Caulobacter crescentus* stalked and swarmer cells. J. Bacteriol..

[54] Caccamo PD, Jacq M, VanNieuwenhze MS, Brun YV (2020). A division of labor in the recruitment and topological organization of a bacterial morphogenic complex. Curr. Biol..

[55] Robertson, N., Sanders, D. P., Seymour, P. & Thomas, R. A new proof of the four-colour theorem. *Electron. Res. Announc. Amer. Math. Soc*. 10.1090/S1079-6762-96-00003-0 (1996).

[56] Hartmann R, van Teeseling MCF, Thanbichler M, Drescher K (2020). BacStalk: a comprehensive and interactive image analysis software tool for bacterial cell biology. Mol. Microbiol..

[57] Goni-Moreno, A., Kim, J. & de Lorenzo, V. CellShape: a user-friendly image analysis tool for quantitative visualization of bacterial cell factories inside. *Biotechnol. J.*10.1002/biot.201600323 (2017). 10.1002/biot.201600323 27492366

[58] Young JW (2011). Measuring single-cell gene expression dynamics in bacteria using fluorescence time-lapse microscopy. Nat. Protoc..

[59] O’Connor, O. M. et al. DeLTA 2.0: a deep learning pipeline for quantifying single-cell spatial and temporal dynamics. *PLoS Comput. Biol.* **18**, 10.1101/2021.08.10.455795 (2021). 10.1371/journal.pcbi.1009797 PMC8797229 35041653

[60] Srivastava N, Hinton G, Krizhevsky A, Sutskever I, Salakhutdinov R (2014). Dropout: a simple way to prevent neural networks from overfitting. J. Mach. Learn. Res..

[61] Kaur H, Pannu HS, Malhi AK (2019). A systematic review on imbalanced data challenges in machine learning: applications and solutions. ACM Comput. Surv..

