# Supplementary material for: Omnipose: a high-precision morphology-independent solution for bacterial cell segmentation
Source: Nat Methods. 2022 Oct 17;19(11):1438–48. doi: 10.1038/s41592-022-01639-4 (PMC9636021; doi:10.1038/s41592-022-01639-4)
Supplement: Supplementary file 1 — Supplementary Table references. [file 41592_2022_1639_MOESM1_ESM.pdf]

nature methods

Article

https://doi.org/10.1038/s41592-022-01639-4

Omnipose: a high-precision morphology-independent solution for bacterial cell segmentation

In the format provided by the
authors and unedited

Supplementary Table References

1 Meberg, B. M., Sailer, F. C., Nelson, D. E. & Young, K. D. Reconstruction of Escherichia coli mrcA (PBP 1a) mutants lacking multiple combinations of penicillin binding proteins. *J Bacteriol* **183**, 6148–6149, doi:10.1128/JB.183.20.6148-6149.2001 (2001).

2 Barbe, V. *et al*. Unique features revealed by the genome sequence of Acinetobacter sp. ADP1, a versatile and naturally transformation competent bacterium. *Nucleic Acids Res* **32**, 5766-5779, doi:10.1093/nar/gkh910 (2004).

3 Yu, Y. *et al*. Genomic patterns of pathogen evolution revealed by comparison of Burkholderia pseudomallei, the causative agent of melioidosis, to avirulent Burkholderia thailandensis. *BMC microbiology* **6**, 46 (2006).

4 Lowenthal, A. C. *et al*. Functional analysis of the Helicobacter pylori flagellar switch proteins. *J Bacteriol* **191**, 7147-7156, doi:10.1128/JB.00749-09 (2009).

5 Evinger, M. & Agabian, N. Envelope-associated nucleoid from Caulobacter crescentus stalked and swarmer cells. *J Bacteriol* **132**, 294-301, doi:10.1128/jb.132.1.294-301.1977 (1977).

6 Allue-Guardia, A., Echazarreta, M., Koenig, S. S. K., Klose, K. E. & Eppinger, M. Closed Genome Sequence of Vibrio cholerae O1 El Tor Inaba Strain A1552. *Genome Announc* **6**, doi:10.1128/genomeA.00098-18 (2018).

7 Stover, C. K. *et al*. Complete genome sequence of Pseudomonas aeruginosa PA01, an opportunistic pathogen. *Nature* **406**, 959–964 (2000).

8 Stringer, C., Wang, T., Michaelos, M. & Pachitariu, M. Cellpose: a generalist algorithm for cellular segmentation. *Nature methods* **18**, 100-106, doi:10.1038/s41592-020-01018-x (2021).

9 Wolny, A. *et al*. Accurate and versatile 3D segmentation of plant tissues at cellular resolution. *Elife* **9**, doi:10.7554/eLife.57613 (2020).
