# Supplementary material for: Omnipose: a high-precision morphology-independent solution for bacterial cell segmentation
Source: Nat Methods. 2022 Oct 17;19(11):1438–48. doi: 10.1038/s41592-022-01639-4 (PMC9636021; doi:10.1038/s41592-022-01639-4)
Supplement: Supplementary file 2 — Reporting Summary [file 41592_2022_1639_MOESM2_ESM.pdf]

nature research

Corresponding author(s): Joseph Mougous

Last updated by author(s): 27/7/2022

Reporting Summary

Nature Research wishes to improve the reproducibility of the work that we publish. This form provides structure for consistency and transparency in reporting. For further information on Nature Research policies, see our Editorial Policies and the Editorial Policy Checklist.

Statistics

For all statistical analyses, confirm that the following items are present in the figure legend, table legend, main text, or Methods section.

n/a	Confirmed	
☐	☒	The exact sample size (*n*) for each experimental group/condition, given as a discrete number and unit of measurement
☐	☒	A statement on whether measurements were taken from distinct samples or whether the same sample was measured repeatedly
☐	☒	The statistical test(s) used AND whether they are one- or two-sided<br>*Only common tests should be described solely by name; describe more complex techniques in the Methods section.*
☒	☐	A description of all covariates tested
☐	☒	A description of any assumptions or corrections, such as tests of normality and adjustment for multiple comparisons
☒	☐	A full description of the statistical parameters including central tendency (e.g. means) or other basic estimates (e.g. regression coefficient) AND variation (e.g. standard deviation) or associated estimates of uncertainty (e.g. confidence intervals)
☐	☒	For null hypothesis testing, the test statistic (e.g. F, t, r) with confidence intervals, effect sizes, degrees of freedom and P value noted<br>*Give P values as exact values whenever suitable.*
☒	☐	For Bayesian analysis, information on the choice of priors and Markov chain Monte Carlo settings
☒	☐	For hierarchical and complex designs, identification of the appropriate level for tests and full reporting of outcomes
☒	☐	Estimates of effect sizes (e.g. Cohen's *d*, Pearson's *r*), indicating how they were calculated

Our web collection on statistics for biologists contains articles on many of the points above.

Software and code

Policy information about availability of computer code

Data collection	Data acquisition for this study was performed using NIS-Elements 4.30.02 or Micro-Manager 1.4.
Data analysis	Data processing and analysis scripts for this study were written in MATLAB 2021a and Python>=3.8.5. All scripts are available at https://github.com/kevinjohncutler/omnipose/tree/main/figures. Figure panels were assembled in Adobe Illustrator v26.2.1. The algorithms used in comparison to Omnipose are sourced as follows: SuperSegger v.1.1: https://github.com/wiggins-lab/SuperSegger. Cellpose v1.0.2: https://github.com/MouseLand/cellpose. StarDist v0.7.2: https://github.com/stardist/stardist. MiSiC v1.0.3: https://github.com/pswapnesh/misic. Mask R-CNN from torchvision v0.12.0: https://github.com/pytorch/vision. Morphometrics v1.102: https://simtk.org/projects/morphometrics.

For manuscripts utilizing custom algorithms or software that are central to the research but not yet described in published literature, software must be made available to editors and reviewers. We strongly encourage code deposition in a community repository (e.g. GitHub). See the Nature Research guidelines for submitting code & software for further information.

Data

Policy information about availability of data

All manuscripts must include a data availability statement. This statement should provide the following information, where applicable:

- Accession codes, unique identifiers, or web links for publicly available datasets
- A list of figures that have associated raw data
- A description of any restrictions on data availability

Bacterial phase contrast and fluorescence image sets generated in this study, unpublished bacterial phase contrast images provided by Yves Brun and unpublished minimum projection brightfield images of C. elegans provided by Luca Rappez are available in our OSF repository https://osf.io/xmury/ under the CC BY-NC 3.0 license. Additional C. elegans images were sourced from a selection of the Open Worm Movement dataset by the Wormpose project at https://github.com/iteal/wormpose_data and from the Broad Bioimage Benchmark Collection set BBBC010 at https://bbbc.broadinstitute.org/c-elegans-livedead-assay-0, both of which are

available under the CC-4.0 license. Files deriving from our processing and labeling of these data are available in our OSF repository https://osf.io/xmury/ under the CC BY-NC 3.0 license. . A. thaliana image volumes were sourced from the PlantSeg OSF database at https://osf.io/uzq3w/. The cyto2 dataset was sourced from http://www.cellpose.org/dataset.

Field-specific reporting

Please select the one below that is the best fit for your research. If you are not sure, read the appropriate sections before making your selection.

☒ Life sciences ☐ Behavioural & social sciences ☐ Ecological, evolutionary & environmental sciences

For a reference copy of the document with all sections, see nature.com/documents/nr-reporting-summary-flat.pdf

Life sciences study design

All studies must disclose on these points even when the disclosure is negative.

Sample size	The principal findings of this study are of cell segmentation performance, and the number of images analyzed and the density of cells within those images were in line with those employed in other cell segmentation studies (Cellpose, StarDist, MiSiC), where the size of the sample size is set by the number of labeled cells in the ground-truth dataset(s). The data collected and labeled for this study was structured specifically to represent the wide range of cell types observed in bacterial phase contrast microscopy that have been the subject of previous efforts to develop bacterial segmentation algorithms (SuperSegger, Morphometrics, MiSiC, BactMAP, BacStalk, Cellprofiler, CellShape, ColiCoords, Cytokit, MicroAnalyzer, MicrobeJ, Oufti, and Schnitzcells) and contains > 40,000 cells. A secondary, experimental finding of this study relates to the analysis of cell phenotypes on unlabeled data (lines 308-335), which has a sample size of ~300,000 cells. This number is 100x greater than the previous study on the same system (https://doi.org/10.1016/j.cell.2018.09.037, Fig. S3).
Data exclusions	Image regions exhibiting overlapping cells/organisms were excluded from our analyses.
Replication	Replication is not relevant to the principal findings of this study of cell segmentation performance, as these results do not vary with repeated evaluation or training runs given the same ground truth data and parameters. The experimental findings related to morphological phenotypes (lines 308-335) constitutes a single biological replicate. This experiment recapitulated (to much higher precision and significance) the results of a previously published biological replicate in our lab (https://doi.org/10.1016/j.cell.2018.09.037, Fig. S3).
Randomization	Ground truth data was partitioned randomly into training and validation sets.
Blinding	Blinding is not applicable to this study because all data and algorithms need to be explicitly defined to run the computational analysis.

Reporting for specific materials, systems and methods

We require information from authors about some types of materials, experimental systems and methods used in many studies. Here, indicate whether each material, system or method listed is relevant to your study. If you are not sure if a list item applies to your research, read the appropriate section before selecting a response.

Materials & experimental systems

n/a	Involved in the study	
☒	☐	Antibodies
☒	☐	Eukaryotic cell lines
☒	☐	Palaeontology and archaeology
☒	☐	Animals and other organisms
☒	☐	Human research participants
☒	☐	Clinical data
☒	☐	Dual use research of concern

Methods

n/a	Involved in the study	
☒	☐	ChIP-seq
☒	☐	Flow cytometry
☒	☐	MRI-based neuroimaging
